# Simulator training in focus assessed transthoracic echocardiography (FATE) for undergraduate medical students: results from the FateSim randomized controlled trial

**DOI:** 10.1186/s12909-024-06564-y

**Published:** 2025-01-04

**Authors:** Johannes Matthias Weimer, Franziska Marietta Sprengart, Thomas Vieth, Sebastian Göbel, Anna Dionysopoulou, Rebecca Krüger, Jan Beer, Andreas Michael Weimer, Holger Buggenhagen, Roman Kloeckner, Lukas Pillong, Johanna Helfrich, Elias Waezsada, Philipp Wand, Julia Weinmann-Menke

**Affiliations:** 1 Rudolf Frey Learning Clinic, University Medical Center of the Johannes Gutenberg University Mainz, Mainz, Germany; 2 Department of Internal Medicine I, University Medical Center of the Johannes Gutenberg University Mainz, Mainz, Germany; 3 Rehabilitation Center Bayerisch Gmain, Bayerisch Gmain, Germany; 4 Department of Obstetrics and Gynecology, University Medical Center of the Johannes Gutenberg University Mainz, Mainz, Germany; 5 Department of Cardiac and Vascular Surgery, University Medical Center of the Johannes Gutenberg University Mainz, Mainz, Germany; 6 Department of Orthopedics, Trauma Surgery and Spinal Cord Injury, Heidelberg Trauma Research Group, Heidelberg University Hospital, Heidelberg, Germany; 7 Institute of Interventional Radiology, University Hospital Schleswig-Holstein - Campus Lübeck, Lübeck, Germany; 8 Department of Otorhinolaryngology, University of Saarland, 66123 Homburg, Germany; 9 Clinic for Electrophysiology, Heart and Diabetes Centre NRW, Ruhr University Bochum, Bad Oeynhausen, Germany; 10 Department of Visceral, Thoracic and Vascular Surgery, University Hospital Carl Gustav Carus, Dresden, Germany

**Keywords:** Simulation-based training, ‌High-Fidelity Simulation, Ultrasound education, FATE, FOCUS, Randomized controlled trials

## Abstract

**Introduction:**

Ultrasound is important in heart diagnostics, yet implementing effective cardiac ultrasound requires training. While current strategies incorporate digital learning and ultrasound simulators, the effectiveness of these simulators for learning remains uncertain. This study evaluates the effectiveness of simulator-based versus human-based training in Focused Assessed with Transthoracic Echocardiography (FATE).

**Materials and methods:**

This single-centre, prospective, randomised controlled study was conducted during an extracurricular FATE workshop (approximately 420 min) for third-year medical students. Participants were randomly assigned to the study group (training solely on simulators) or the control group (training on human subjects). Both groups completed a theory test and a self-assessment questionnaire before the course (T_1_) and at the end of the training (T_2_). At T_2_, all participants also completed two Direct Observation of Procedural Skills (DOPS) tests—one on the simulator (DOPS^Sim^) and one on humans (DOPS^Human^).

**Results:**

Data from 128 participants were analysed (*n* = 63 study group; *n* = 65 control group). Both groups exhibited increased competency between the T_1_ and T_2_ self-assessments and theory tests (*p* < 0.01). In the DOPS^Human^ assessment at T_2_, the control group performed significantly better (*p* < 0.001) than the study group. While motivation remained consistently high among both groups, the study group rated their “personal overall learning experience” and the “realistic nature of the training” significantly worse than the control group (*p* < 0.0001). Both groups supported the use of ultrasound simulators as a “supplement to human training” (study: 1.6 ± 1.1 vs. control: 1.7 ± 1.2; *p* = 0.38), but not as a “replacement for human training” (study: 5.0 ± 2.3 vs. control: 5.4 ± 2.1; *p* = 0.37).

**Conclusion:**

Both simulator- and human-based training effectively developed theoretical and practical skills in FATE. However, the simulator group demonstrated significantly poorer performance when applying their skills to human subjects, indicating limitations in the transferability of this simulator-based training to real-life patient care. These limitations of simulator-based ultrasound training should be considered in future training concepts.

**Clinical trial number:**

Not Applicable.

**Supplementary Information:**

The online version contains supplementary material available at 10.1186/s12909-024-06564-y.

## Introduction

Examination of the heart using ultrasound is an important diagnostic method in cardiology [1]. Cardiac ultrasound is recommended in nearly all cardiac guidelines due to its ready availability, speed of implementation, and cost-effectiveness [1]. Nevertheless, achieving standardised and diagnostically reliable echocardiographic imaging can be challenging [2], requiring thorough training to ensure safety, accurate technique, and proper documentation [3, 4]. Basic skills are initially developed in training courses and reinforced through numerous supervised echocardiographic examinations, as outlined in the certification requirements of various professional societies [5].

Recently, specialised training concepts for students have been introduced at universities as part of medical degree programs [6–12], allowing students to acquire basic skills in performing echocardiography or focused sonography of the heart (FocUs) at an early stage [8]. This reflects the advice of international professional societies, which advocate for early training of students in ultrasound diagnostics and provide guidelines for its integration into education [13].

The COVID-19 pandemic, with its social distancing measures, further increased the demand for innovative, digitally supported teaching methods such as blended learning and simulator-based training [5, 14–25]. A wide range of ultrasound simulators are now commercially available, varying in their technical implementation, areas of diagnostic application, and the types of simulation images displayed on the viewing screen, which range from real CT and ultrasound images of patients to computer-generated images [21, 26].

Ultrasound simulators are frequently evaluated for their usefulness and applicability in training [7, 23, 27–34], particularly as an alternative or supplement to practising on live subjects [7, 14, 15, 17, 22, 23, 35–43]. Simulator-based training has shown benefits, particularly in improving diagnostic accuracy during patient examinations [28, 30].

Although evidence supporting the transfer of theoretical and practical skills from simulators to real patient care exists [44–47], larger randomized trials directly comparing simulator-based training with live-subject training for echocardiography skill transfer among medical students remain lacking, and current results vary significantly [23, 30, 42, 48]. This heterogeneity also extends to studies on simulation-based echocardiography training [7, 14, 36–38, 42, 49–51]. This study addresses these gaps by investigating how theoretical and practical skills in Focused Assessed Transthoracic Echo (FATE) are developed through simulator-based training compared to training on live subjects, and how effectively these skills translate to real patient examinations.

The goal of this study is to better understand the differences in training effectiveness in echocardiography, ultimately improving ultrasound education. Based on this randomised, prospective study, we propose that students’ ability to apply their training to real patients differs depending on whether they were trained using simulators or live subjects.

## Materials and methods

### Study design

**Fig. 1:**
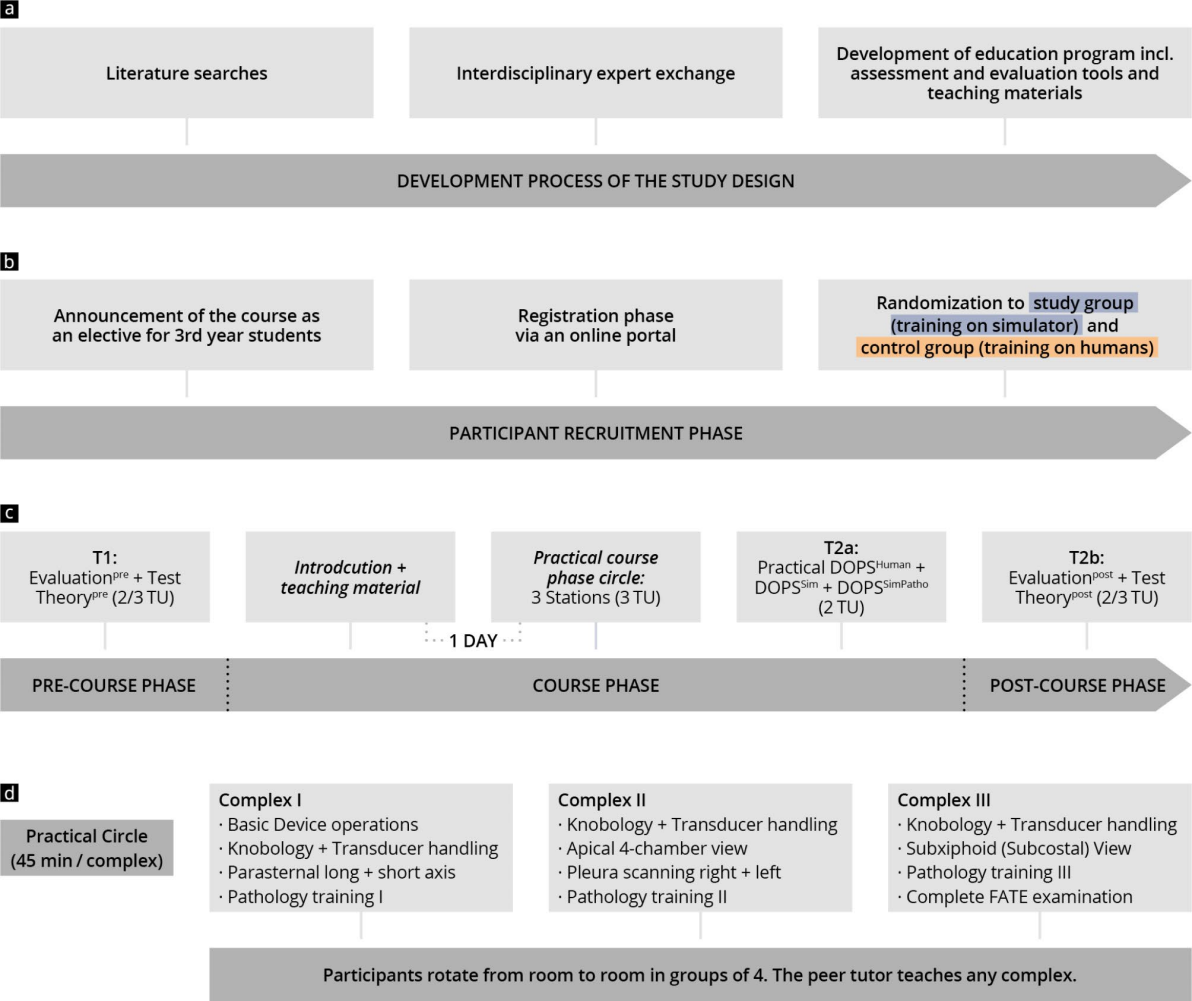
Chronological presentation of the study design and course concept, including data collection times (T_1_, T_2a,_ and T_2b_). (**a**) The development process of the course design and content; (**b**) recruitment; (**c**) course concept; (**d**) course modules including learning objectives

This single-centre, prospective randomised controlled study (Fig. 1) was designed and conducted at a Capitalize University Hospital in 2022 [52, 53].

This study utilized a validated ultrasound simulator (Vimedix, CAE Healthcare, Sarasota, Florida, US), approved by experts [54], in voluntary extracurricular workshops aimed at teaching the FATE protocol [55]. This was compared to traditional training using live human subjects. The 3rd year of the undergraduate medical degree programme corresponds to the first year of clinical training. Only third-year students were included to ensure that participants had no prior experience in echocardiography. The students were invited to participate in the study via an official announcement distributed to a mailing list from the Capitalize Dean’s Office. Volunteers registered for the workshops via an online portal.

After the enrolment deadline, participants were randomly assigned to either the study group (training on the simulator) or the control group (training on human subjects). Assessments, including self-evaluations (Evaluation^pre^, Evaluation^post^), theory tests (Theory^pre^, Theory^post^), and practical exams (DOPS^Sim^, DOPS^SimPatho^, DOPS^Human^), were conducted at two time points (T1 = pre and T2a and T2b = post) [56, 57]. Inclusion criteria required participants to have completed the first state examination, provided informed consent to participate in the study, and fully attended the introductory event, workshop, and examinations.

The primary endpoints of the study are threefold: an improvement in competency, as measured by theory tests; an improved practical skill level, as assessed by a practical FATE examination on either a simulator or live subject; and an ability to detect pathology. Secondary endpoints include a subjective increase in competency and motivation; the subjective achievement of the defined learning objectives; and an evaluation of the training concept.

### Course concept and learning objectives

A FATE-specific workshop [55] was developed based on a training concept for focused sonography of the heart (FOCUS) [8] utilizing cross sections in transthoracic echocardiography [58], as proposed by the World Interactive Network Focused on Critical Ultrasound (WINFOCUS). The 360-minute workshop included an introductory session in plenary (90 min), practical exercises in small groups with practical tests (225 min), and a final plenary session (45 min). The learning objectives and the developed module sequence are presented in Fig. 1 and Supplement 1.

During the introductory session, held one day before the practical part of the workshop, participants completed a theory test (Theory^pre^) and an initial Evaluation^pre^. These were followed by a brief guided tour of the FATE protocol through a live pre-workshop session, and a study poster was distributed. During the practical exercises, participants rotated through 3 stations (135 min), each focussing on orientation in cross-sections and pathology training. Within the stations the control group was shown pre-recorded real ultrasound clips of pathologies, while the study group practiced case scenarios of pathologies on the simulator. To ensure uniformity in meeting the learning objectives, detailed instructions and station tasks for small group teaching were developed beforehand for each station (see Supplements 2 and 3).

At the end of the workshop, all participants completed the same Direct Observation of Procedural Skills test (90 min) on both the simulator (DOPS^Sim^, DOPS^SimPatho^) and real people (DOPS^Human^). They then took a theory post-test (Theory^post^) and self-evaluation (Evaluation_post_) in plenary (45 min). The control group first completed the tests on humans then on the simulator, the study group vice-versa.

A total of six workshops were held, each with six stations (three for the control group and three for the study group) running in parallel. Four grouped students per station were supervised by one tutor.

### Tutors and equipment

A total of ten didactically and professionally trained peer tutors [8] (i.e., students from clinical semesters) taught the participants during the workshop under the supervision of two consultants. All tutors underwent systematic training on the simulator to master its operating functions.

**Fig. 2:**
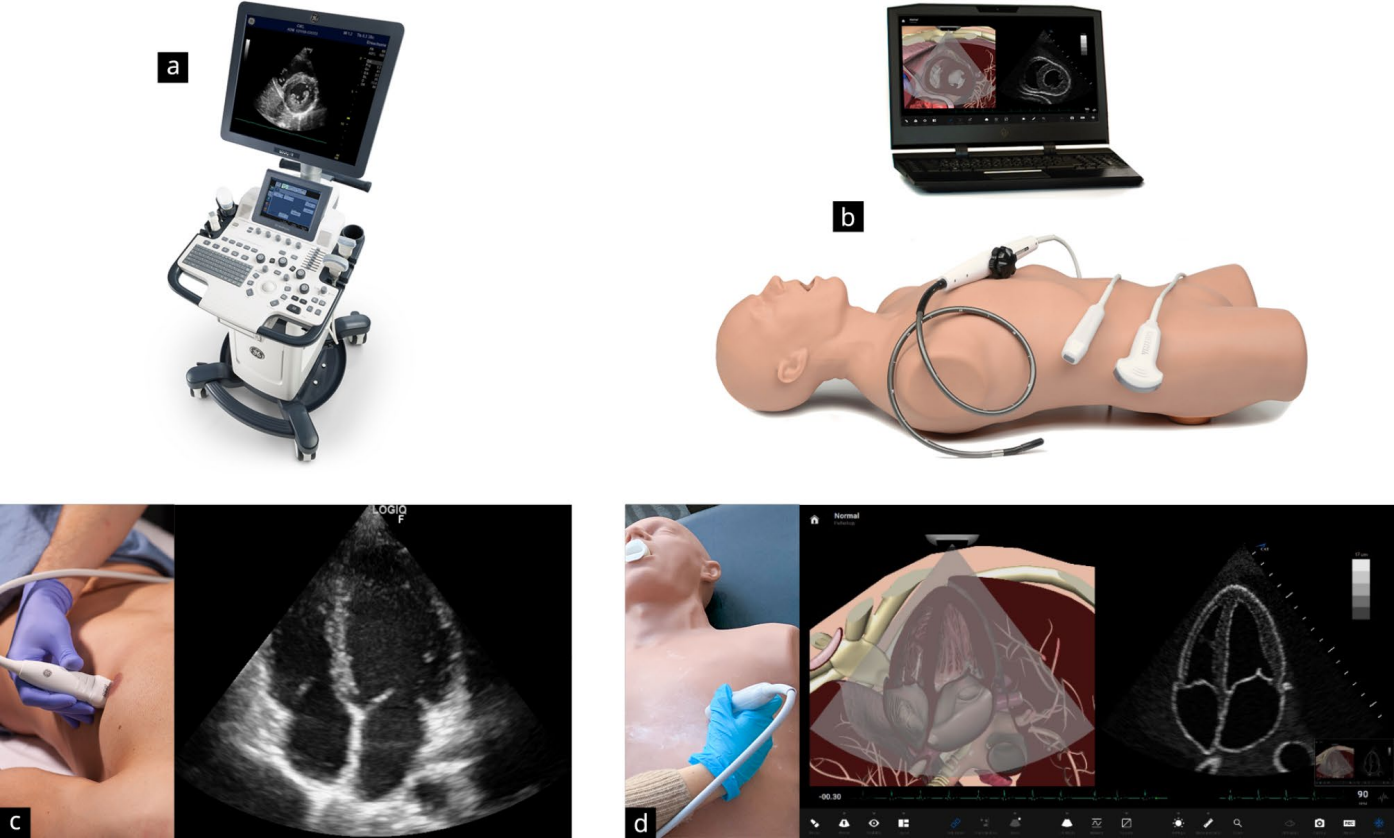
Presentation of the training equipment used in the course. The control group trained on the ultrasound system GE F8 by General Electric Company, Boston (Fig. 2**a** and **c**), and the study group trained on the ultrasound simulator Vimedix by CAE Healthcare, Sarasota, Florida, US (Fig. 2**b** and **d**)

A total of three ultrasound devices from GE HealthCare (GE F8; General Electric Company, Boston) and three simulators (Vimedix, CAE Healthcare, Sarasota, Florida, US) were used (see Fig. 2). The ultrasound simulators have a life-sized sector transducer that can be applied realistically to a human torso model. The system provides a wide range of cardiology training sets and simulates an array of pathologies (see Supplement 4). In addition to displaying an anatomical cross-sectional image via animation, the monitor also projects an animated ultrasound image. Various measurement tools, Doppler functions, and other device functions can be used realistically. Additionally, transoesophageal echocardiography can be practised by changing the ultrasound probe.

### Assessments

The test design and evaluation instruments used are based on the consensus of ultrasound experts, instructors, and current professional recommendations [56–59].

### Questionnaires

Evaluation^pre^ and Evaluation^post^ (both approximately 5 min) addressed various topics across multiple items. These included “personal data”, “previous experience”, “simulator usage”, “motivation/expectations”, “learning goals”, “subjective competency assessment”, “course evaluation”, “teaching material”, and “tutor evaluation”. The answers were recorded using a seven-level Likert answering format (1 = strongly agree; 7 = strongly disagree), or by dichotomous questions (“yes”/“no”) and free text.

### Pre- and post-test

The theory test (max. 74 points in Theory^pre^ and max. 83 points in Theory^post^), comprised the competency areas “anatomy” (max. 11 points); “basic principles” (max. 14 points); “normal findings = section assignments” (max. 10 points); “section labelling = normal findings or structure recognition in orientation sections” (50 points); and “pathology (recognition)” (max. 9 points, exclusively at T_2_), each derived from the learning objectives. The test comprised labelling, fill-in-the-blank, and multiple-choice question types [56, 60] (see Supplement 5 for example questions). The processing time per test was 25 min.

### Practical tests (DOPS)

Practical skills were assessed by DOPS adapted from previous studies [61]. The DOPS tests, with a processing time of 10 min each, were carried out on the simulator (DOPS^Sim^, max. 78 points) and human test subjects (DOPS^Human^, max. 78 points). A total of six standard cross-sections of the FATE protocol [55] were tested as part of a case study (see Supplement 6). The test subjects were voluntary students, all of whom had a similar BMI.

The competency areas “communication” (max. 8 points); “transducer handling/device operation/patient guidance” (max. 14 points); “examination procedure FATE 1–6” (max. 36 points); “image explanation FATE 1–6” (max. 12 points); and “overall performance” (max. 8 points) were assessed in the tests.

Subsequently, a total of 4 case scenarios were completed on the simulator in the DOPS^SimPatho^ (total of max. 48 points; total processing time 10 min) to assess pathology recognition (see Supplement 7). The competency areas “examination procedure or skill” (2 points); “pathology recognition” (2 points); and “overall impression” (8 points) were assessed in each case.

### Statistics

Data for the evaluations as well as theoretical and practical learning success checks were manually evaluated using Microsoft Excel before analysis in R studio (RStudio Team [2020]. RStudio: Integrated Development for R. RStudio, PBC, http://www.rstudio.com, last accessed 09 02 2024) with R 4.0.3 (A Language and Environment for Statistical Computing, R Foundation for Statistical Computing, http://www.R-project.org; last accessed 09 02 2024). Binary and categorical baseline parameters are expressed as absolute numbers and percentages. Continuous data are expressed as median and interquartile range (IQR) or mean and standard deviation (SD). Categorical parameters were compared using the chi-squared test and continuous parameters using the Mann-Whitney test. In addition, pairwise correlations of metric variables were obtained, and the correlation effect sizes and significances were calculated for both groups. Then, the Fisher z-transformation was used to compare correlations between the two groups. Finally, a multivariate linear regression model was employed to compare the influence of individual factors (“participation in an abdominal ultrasound course”, “lready had contact with simulator-based training”, “already had contact with ultrasound simulators”, “Number of independent sonographic examinations”, “Number of independent echocardiographies”, membership of the control group). *P*-values < 0.05 were considered statistically significant. A power analysis was conducted for this study to determine the sample size required to detect a statistically significant effect. Based on an expected effect size of 0.6, a significance level of 0.05, and a desired power of 0.90, the calculated sample size was set at 120 participants.

## Results

### Baseline

A total of 128 students were included in the study, with 63 in the study group and 65 in the control group (see Supplement 8). The baseline characteristics of both groups were similar (see Table 1), with both having almost equivalent demographic characteristics and prior training profiles. Both groups had a similar average age (study: 24 ± 4 years vs. control: 25 ± 4 years), were mainly female (study: 67% vs. control: 62%), and most had not previously used ultrasound simulators (study: 97% vs. control: 91%). Most had already taken an abdominal sonography course (study: 57% vs. control: 57%) but had not performed any independent echocardiograms (study: 95% vs. control: 85%; *p* = 0.02).

**Table 1:** Group statistics at baseline

Item	Parameter	Control group	Study group	*p*-value
Age	Years (mean ± SD)	25 ± 4	24 ± 4	0.19
Sex	Male (n)	25	21	0.67
	Female (n)	40	42	
Prior education	yes (n)	36	24	0.07
	no (n)	29	39	
University radiology course	n	1	3	0.59
Course in abdomen sonography	n	37	36	1.0
Number of observed	none	6	5	0.37
sonographic examinations	<=5	1	0	
	< 20	41	49	
	20–40	9	5	
	>= 40	8	4	
Number of independent sonographic examinations	none	12	9	0.62
	< 20	51	52	
	20–40	1	2	
	>= 40	1	0	
Number of independent echocardiographies	none	55	60	0.02
	< 20	9	1	
	20–40	1	0	
Prior simulator training	n	28	33	0.38
Prior use of ultrasound simulator	n	6	2	0.29

### Motivation, learning objectives, simulators and course concept

**Fig. 3:**
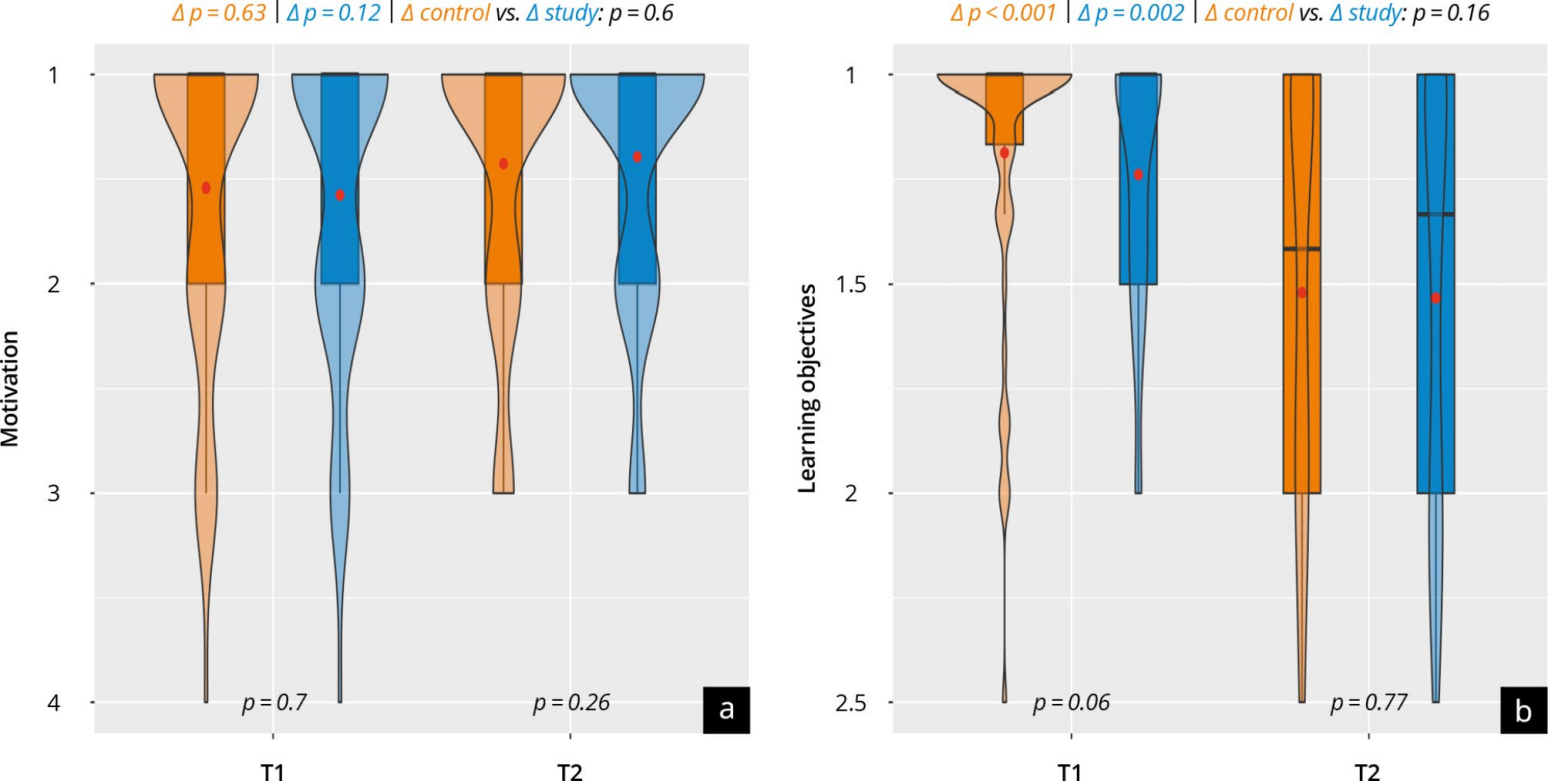
Self-evaluation of (**a**) motivation and (**b**) achievement of learning objectives at time points T1 and T2. The control group are represented in orange and the study group in blue

The evaluation of personal motivation, achievement of learning objectives, and the course concept are shown in Fig. 3 + 4 and Supplement 9 + 10. Both groups were similarly positively motivated at T_1_ and T_2_ (scale point range 1.4–1.6). Most participants in both groups state they achieved the learning objectives of the course overall and per subitem (scale point difference T_1_-T_2_: 0.2-04). No significant differences were found in “personal satisfaction and benefit of the course” (study: 2.7 ± 0.7 vs. control: 2.4 ± 1.0; *p* = 0.97) or “overall course evaluation” (study: 2.2 ± 0.9 vs. control: 2.4 ± 0.9; *p* = 0.09). The overall tutor evaluation was significantly more positive in the study group, with both groups (study: 1.4 ± 0.7 vs. control: 1.7 ± 0.7; *p* = 0.03) evaluating the tutors in very good scale ranges. The study group rated their subjective “personal overall learning experience” significantly worse than the control group (study: 2.3 ± 0.9 vs. control: 1.8 ± 0.7; *p* < 0.0001), which mainly results from the significantly worse assessment of the “realism of the training” (study: 3.5 ± 1.6 vs. control: 1.7 ± 0.9; *p* < 0.0001). Both groups support the use of an ultrasound simulator for training purposes as a “supplement to training on humans” (study: 1.6 ± 1.1 vs. control: 1.7 ± 1.2; *p* = 0.38), but not as a “replacement for training on humans” (study: 5.0 ± 2.3 vs. control: 5.4 ± 2.1; *p* = 0.37).

**Fig. 4:**
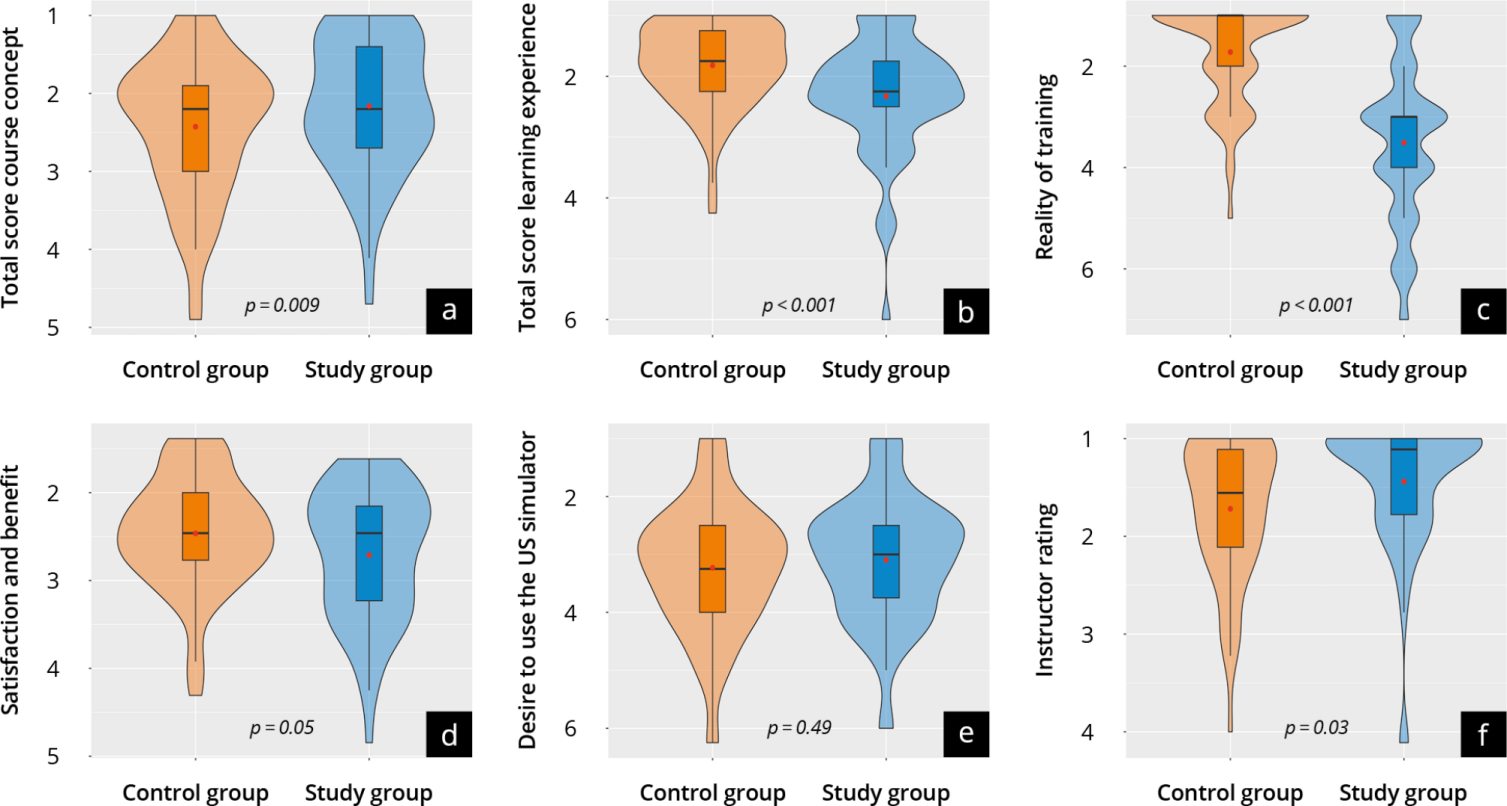
Evaluation results at T_2_ with the control group represented in orange and the study group represented in blue. (**a**) Results of the evaluation of the course concept; (**b**) the learning experience; (**c**) the realism of training; (**d**) the satisfaction and benefit; (**e**) the desire to use the ultrasound simulator; (**f**) the instructor rating

### Subjective gain in competencies

**Fig. 5:**
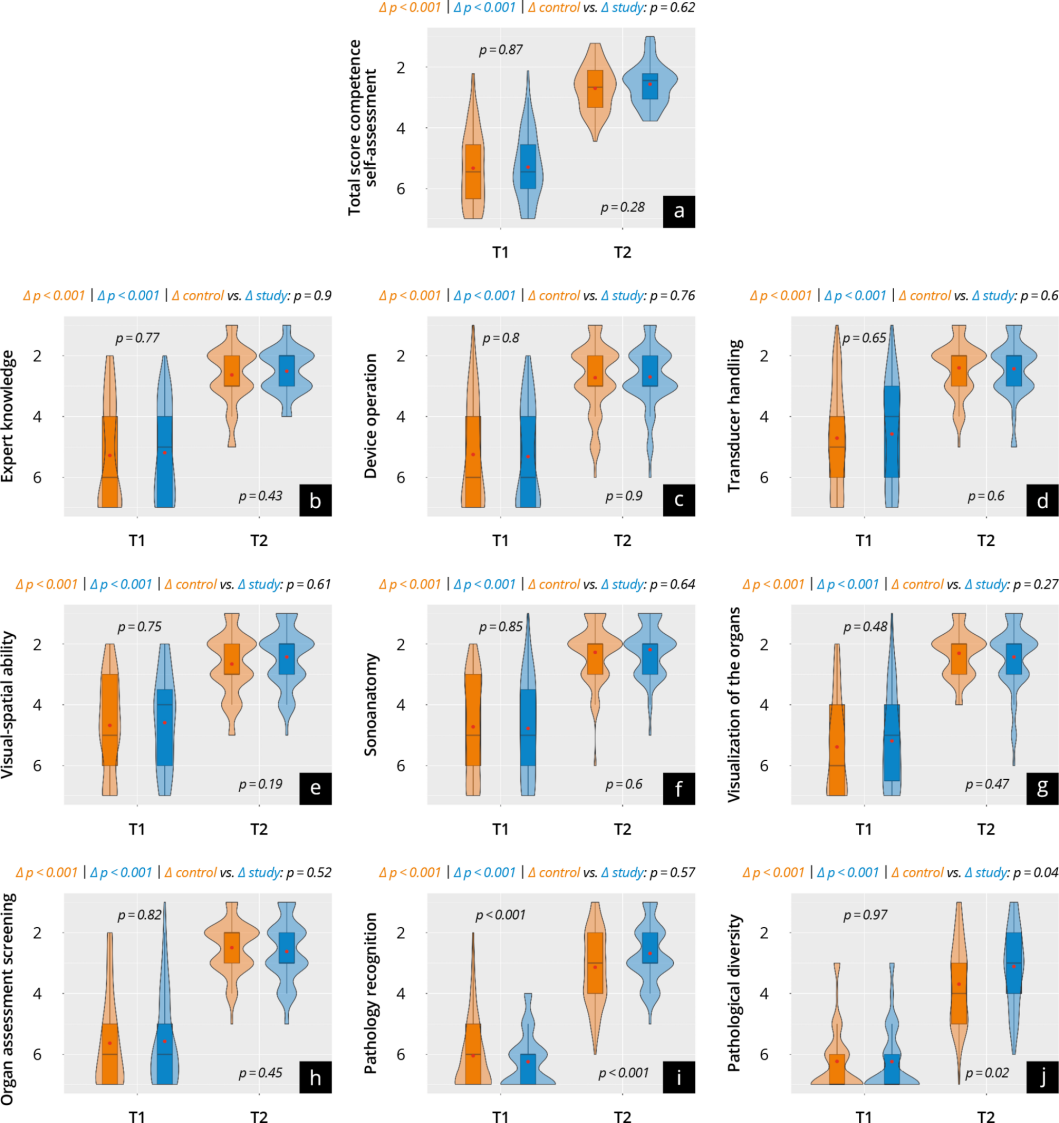
Results of subjective competence development at time points T1 and T2, with the control group represented in orange and the study group in blue. (**a**) The total score; (**b**–**j**) the subitems

The results of the evaluation at T_1_ and T_2_ are shown in Fig. 5 and Supplement 11. No significant differences were found in the overall score at T_1_ (study: 5.3 ± 1.1 vs. control: 5.3 ± 1.2; *p* = 0.87). Both groups reported a significant increase in competency up to T_2_ (Delta *p* < 0.001) and reached a similarly high self-reported competency (study: 2.6 ± 0.7 vs. control: 2.7 ± 0.7; *p* = 0.23). This trend applied to almost all subcategories except for “pathology recognition”. Here, the study group reported a significantly higher increase in competency than the control group (study: Δ 3.7 ± 1.2 vs. control: Δ 2.8 ± 1.8; *p* < 0.01).

### Objective gain in competencies

#### Theory test

**Fig. 6:**
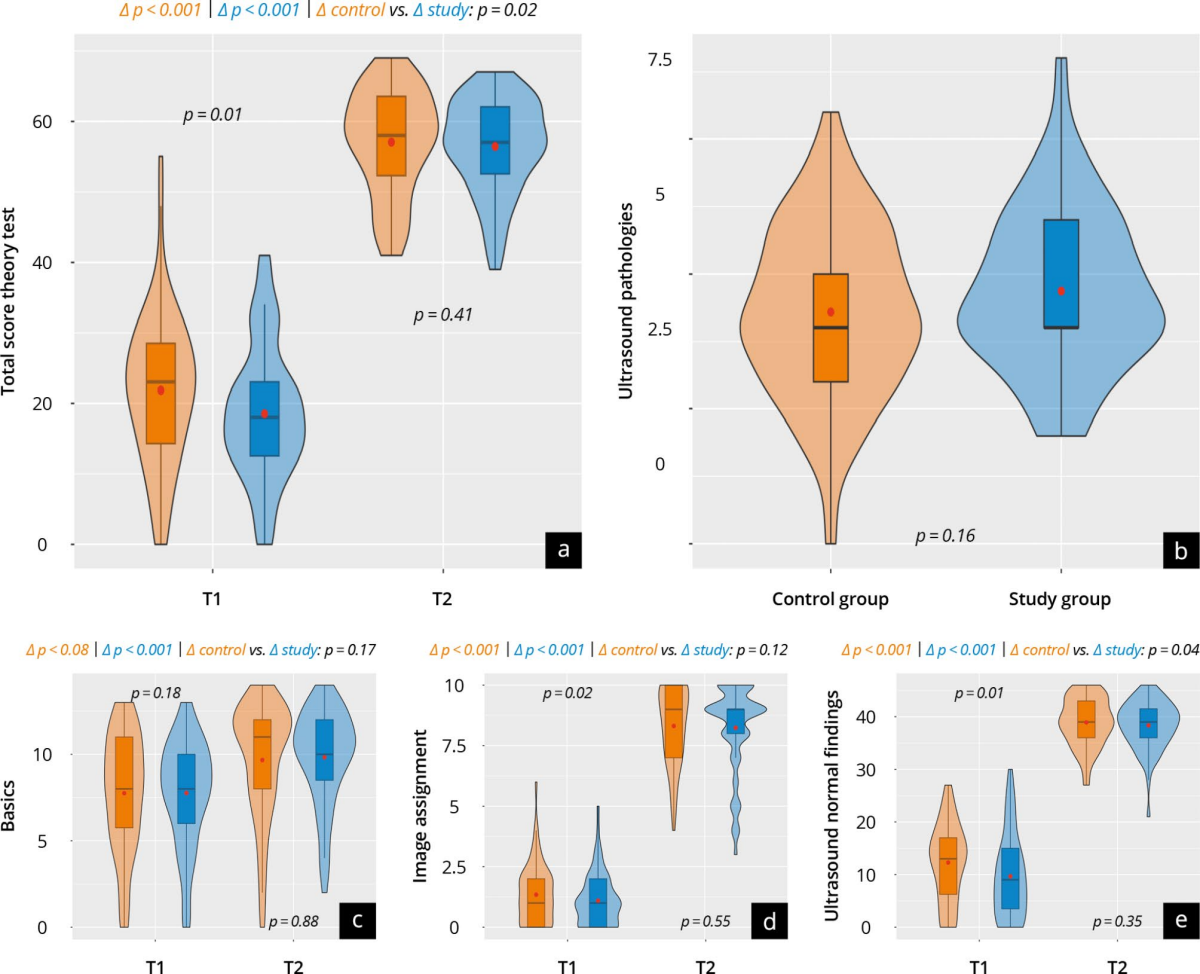
Results of the theory test at T_1_ and T_2b_ with the control group represented in orange and the study group in blue. (**a**) The total score; (**b**) the subcategories ultrasound pathologies; (**c**) basics; (**d**) image assignment; (**e**) ultrasound normal findings

The results of the theory tests at T_1_ (Theory^Pre^) and T_2b_ (Theory^Post^) are shown in Supplement 12 Fig. 6. In the overall score, the control performed better at T_1_ than the study group (study: 19 ± 10 vs. control: 24 ± 16; *p* = 0.01). Throughout the test, both groups achieved a significant (*p* < 0.001) objective increase in competency and demonstrated a similar level of competency at T_2_ (study: 56 ± 7 vs. control: 57 ± 8; *p* = 0.41), with the study group achieving a significantly higher increase (study: Δ 38 ± 9 vs. control: Δ 33 ± 14; *p* = 0.02). In the pathologies examined at T_2_, no significant differences between the groups were found (study: 5 ± 2 vs. control: 4 ± 2; *p* = 0.16).

#### Practical test

**Fig. 7:**
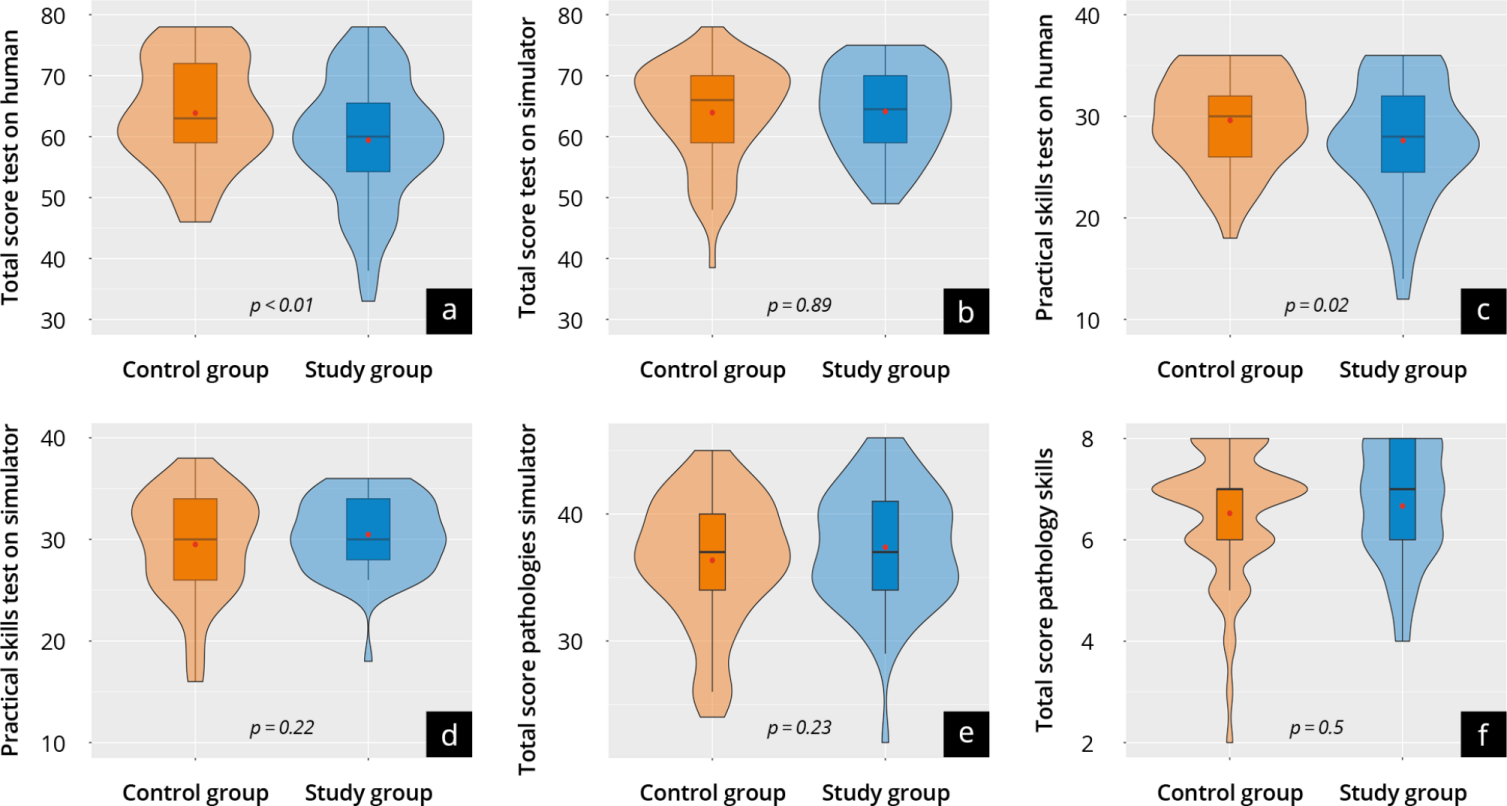
Results of the practical test at time T_2a_ of the control group (orange) and study group (blue). The violin plots present the results of: (**a, d**) DOPS^Human^; (**b, c**) DOPS^Sim^; (**e, f**) DOPS^SimPatho^

The results of the practical tests DOPS^Sim^ and DOPS^Human^ at time T_2_ are shown in Supplement 13 and Fig. 7. Overall, both groups scored well in the DOPS^Sim^ (study: 64 ± 7 vs. control: 64 ± 8; *p* = 0.89) and in the DOPS^SimPatho^ (study: 37 ± 5 vs. control: 36 ± 5; *p* = 0.23). The control group achieved significantly better results in the DOPS^Human^ (study: 59 ± 10 vs. control: 64 ± 9; *p* < 0.01). These trends hold for the subcategories of the respective DOPS, especially regarding “device operation”. When comparing results in DOPS^Sim^ with DOPS^Human^, the control group achieved equivalent results in both DOPS (*p* = 0.97), whereas the study group showed significantly worse results in the DOPS^Human^(*p* < 0.01).

#### Influencing factors and correlations

The multivariable linear regression analysis of the theory tests and practical examinations yielded several influencing factors. In the T1 theory test (Theory^Pre^), these factors were “participation in an abdominal ultrasound course” (β = 6.51; *p* = 0.004); “already had contact with simulator-based training” (β = 5.13; *p* = 0.019); “already had contact with ultrasound simulators” (β = 13.51; *p* = 0.003); and “membership of the control group” (β = 4.24; *p* = 0.048). In the T2 theory test (Theory^Post^), the factor “already had contact with ultrasound simulators” (β = 3.52; *p* = 0.004) had a significant influence. In the linear regression analysis of DOPS^Sim^, DOPS^SimPatho^_,_ and DOPS^Human^, only DOPS^Human^ was significantly influenced by “membership of the control group” (β = 4.76; *p* = 0.005), with no other influencing factors detected.

The correlations between the objective test results of DOPS^Human^ and DOPS^Sim^, DOPS^Human^ and Theory^Post^, DOPS^Human^ and DOPS^SimPatho^ were significantly higher in the control group than in the study group (*p* < 0.03).

## Discussion

### Summary of Key findings + relevance of research

This study compares the effectiveness of simulator-based ultrasound training to traditional ultrasound training with human subjects for teaching theoretical and practical skills in Focused Assessed Transthoracic Echo. It is the first simulator-focussed randomized echocardiographic study to examine an entire semester of students during the clinical phase of a medical degree program. Our results demonstrate that both training approaches can lead to a significant increase in skills, but those trained on a simulator alone did not perform comparably well when performing examinations on real humans. Additionally, participants reported that ultrasound simulators in training could “supplement” training on humans, but they did not accept it as a “replacement”. The results provide important insights into the potential advantages, disadvantages, and challenges of using ultrasound simulators in medical education and offer a basis for future training strategies.

### Gain in competencies

The effectiveness of simulation-based training is still under research, especially regarding the transfer of skills from simulator training to real patient care in cardiac ultrasound diagnostics [17, 18, 20–22, 38, 42, 48, 62]. Preliminary studies have demonstrated that ultrasound simulators promote theoretical [7, 14–16, 21, 30, 31, 33, 34, 36–38, 41–43] and practical [7, 14, 16, 21, 30, 31, 34, 36–38, 41, 43] skill acquisition. These studies also suggested that simulation training was at least equivalent in effectiveness to lecture-based education [16, 33], textbook learning [32], video-based training [15], or e-learning [21, 30, 36]. Simulators offer a forgiving environment that allows early learners to make and learn from errors without the risk of causing harm, thereby fostering a safe and effective training experience. The participants in our study were also able to build theoretical and practical skills in FATE through simulator training, and they reported a subjective increase in their skills. The subjective and objective theoretical skill levels of both study groups at the end of the training were comparable and high [14, 29, 31, 33, 37, 38, 49]. Interestingly, the study group (those training with a simulator) achieved a significantly higher objective theoretical skills increase (T_1_ to T_2_) compared to the control group (training on humans). This indicates that simulator-based training can effectively support the transfer of theoretical knowledge.

While the groups performed nearly identically well in the practical tests on the simulator, the study group performed significantly worse than the control in the test on human subjects. This suggests that training on humans is irreplaceable at the moment and offers a realistic environment that cannot be fully reproduced by current simulators [63]. This finding is consistent with some previous studies [7, 41], but contrasts with other preliminary studies [14, 36–38, 42]. Whereas previous studies assessed the practical competency achieved either on a simulator [36] or human subjects [7, 14, 32, 37, 38, 41, 42], the multiple tests implemented in our study enabled us to make specific statements regarding the groups’ overall and relative development of skills, and, most importantly, regarding the skill transfer between simulator-based training and real human-subject examinations. The comparative results of the practical assessments on humans differed from those of previous findings [14, 36–38]. These differences could be explained by the longer duration of our training concept in comparison with former studies, which would enable finer differences between trained groups to emerge. The inclusion of a much larger number of participants and the use of multiple dedicated testing tools [57] might also explain the differences in our findings and serve to underline the robustness of the data collected.

### Attitude towards simulation, motivation and evaluation of course

Various user groups have demonstrated a positive attitude toward simulators [19, 32, 36, 38, 39, 64]. Our study supports the use of an ultrasound simulator for training purposes as a supplement to training on humans. The participants showed a high level of motivation for training before and after the course, with no decrease in motivation despite the training modality [65]. However, participants across all groups did not view ultrasound simulator training as a replacement for human-based training, which aligns with recommendations from professional associations and preliminary studies [18, 19, 39]. The study group gave a significantly poorer subjective assessment of the overall learning experience and the realism of the training, further emphasizing the need for training on real humans [19, 23, 63]. The general acceptance of this broader training concept [6, 8, 9, 66] supports the future use of a combination of teaching methods. This could eliminate the observed gap in transferring skills from simulator training to real-patient practice, or make the training experience more realistic, thus providing the best possible learning environment [19, 40]. A practical strategy to achieve this would be the implementation of a longitudinal blended learning concept [67], which should include simulator self-learning programmes in paired teams [68] and practical training on humans. Furthermore, other innovative teaching strategies, such as artificial intelligence, virtual reality and telemedicine, could be integrated into ultrasound training in order to facilitate multimodal training [69].

### Strengths and limitations of the study

The strengths of this study include its randomized design, clearly defined teaching methods, and consistent multiple assessment criteria, all of which provide an objective basis for interpreting the results.

However, there are also limitations. These include the voluntary nature of participation, the absence of a control group that received no training, and the reliance on ultrasound simulator models from only one manufacturer. In addition, the study did not explicitly assess the impact of the training modalities on patient safety or quality of care, although previous research suggests that simulator training improves the efficiency of care and reduces patient discomfort and the need for repeated examinations and trainee supervision [19].

A specific economic cost-benefit analysis was also not conducted [70]. While this can be seen as a limitation, certain benefits of simulator-based ultrasound training—such as preparing students for clinical practice, improving patient care and safety, and increasing satisfaction with the educational experience—cannot easily be quantified in monetary terms. Moreover, the study focuses mainly on quantitative assessments of knowledge and practical skills and may overlook qualitative aspects such as learning style preferences.

Despite accounting for several influencing factors through multivariate regression analysis, other personal factors of the participants that were not captured by the evaluations may have influenced the results. Additionally, the different sequencing of the tests (the study group completed the practical test on the simulator first and then on the human model; the control group did it the other way round) could also have influenced the results in ways we could not measure.

Finally, the study’s emphasis on the immediate effects of simulator-based training compared to human-model training does not consider long-term retention of skills. Future studies should explore how well these competencies are maintained over time to better evaluate the effectiveness of training methods [15, 36–38].

## Conclusion

This study enhances our understanding of the effectiveness of approaches to ultrasound teaching. Ultrasound simulators offer promising opportunities, especially in transferring theoretical knowledge to focussed practice of basic skills. Nevertheless, hands-on training with human subjects remains indispensable for effective competency development, supporting the need for future multimodal training strategies. The early implementation of such innovative ultrasound training programs into medical degree curricula and specialist training would improve the quality of medical education and, ultimately, patient care.

## Electronic supplementary material

Below is the link to the electronic supplementary material.

Supplementary Material 1

Supplementary Material 2

Supplementary Material 3

Supplementary Material 4

Supplementary Material 5

Supplementary Material 6

Supplementary Material 7

Supplementary Material 8

Supplementary Material 9

Supplementary Material 10

Supplementary Material 11

Supplementary Material 12

Supplementary Material 13

## Data Availability

Data cannot be shared publicly because of institutional and national data policy restrictions imposed by the Ethics committee since the data contain potentially identifying study participants’ information. Data are available upon request from the Johannes Gutenberg University Mainz Medical Center (contact via weimer@uni-mainz.de) for researchers who meet the criteria for access to confidential data (please provide the manuscript title with your enquiry).

## Declarations

### Authors’ information

J. M. W. is resident in internal medicine and coordinator of the student ultrasound education program of the University Medical Center Mainz. He has more than 10 years of experience in the didactic field and has also worked on the topic of ultrasound training methods in his doctoral thesis. He is a member of the DEGUM, EFSUMB and GMA.
