# Supplementary material for: Simulator training in focus assessed transthoracic echocardiography (FATE) for undergraduate medical students: results from the FateSim randomized controlled trial
Source: BMC Med Educ. 2025 Jan 4;25:21. doi: 10.1186/s12909-024-06564-y (PMC11699650; doi:10.1186/s12909-024-06564-y)
Supplement: Supplementary file 1 — Supplementary Material 1 [file 12909_2024_6564_MOESM1_ESM.pdf]

**Supplement 1.** Learning objectives in the course model developed and adapted from Greim et al. and Weimer et al.

| Topic | Learning objectives: upon completing this course, the participant will be able to: |
|---|---|
| Basic anatomy and physiology | ✓ Describe anatomic relationships in the chest<br>✓ Show the exact position of the heart and the positional relationship of the chambers to each other<br>✓ Explain the physiology of blood flow and valve mechanics during systole and diastole<br>✓ Explain the physiology of blood flow and valve mechanics during systole and diastole<br>✓ Explain the movement of the diaphragm during inhalation and exhalation |
| Physical principles, choice of transducer, and instrument settings | ✓ Explain the physical principles of image acquisition and ultrasound diagnosis<br>✓ Explain the differences between the various transducers and justify the choice of transducer<br>✓ Explain how penetration depth, image resolution, and frequency relate to each other |
| Patient positioning and transducer movement | ✓ Carry out positioning variations and explain how positioning improves the ultrasound conditions<br>✓ Understand and implement defined movement sequences (tilting, angulating, rotating, shifting, swiveling) in relation to image morphology |
| Examination | ✓ Adjust the defined standard sections and explain the plane<br>✓ List relevant questions that can be answered by setting the respective plane<br>✓ Describe the possible transducer movements and adjustable settings available to optimize the planes |

| | |
|---|---|
| Pathologies | ✓ Identify the most common pathologies in in the standard sections: Hyper- and hypovolemia, dilated cardiomyopathy, right heart strain, pericardial effusion/tamponade, mitral valve insufficiency, aortic valve stenosis, left heart hypertrophy, myxoma, thrombus, infarction, pulmonary hypertension, reduced left ventricular pump function, pleural effusion |
