# Supplementary material for: Simulator training in focus assessed transthoracic echocardiography (FATE) for undergraduate medical students: results from the FateSim randomized controlled trial
Source: BMC Med Educ. 2025 Jan 4;25:21. doi: 10.1186/s12909-024-06564-y (PMC11699650; doi:10.1186/s12909-024-06564-y)
Supplement: Supplementary file 2 — Supplementary Material 2 [file 12909_2024_6564_MOESM2_ESM.pdf]

Station 1: Introduction to the devices + parasternal long axis view + parasternal short axis view

☑		Scanning guide	Tips for the examiners
☐	**TN 1**	Obtain the parasternal long axis view of the heart. Orient yourself on the sternoclavicular joint and place the transducer in a way, so that the marker points to the right shoulder (10 o'clock). Move the transducer caudally until the heart is visible.<br>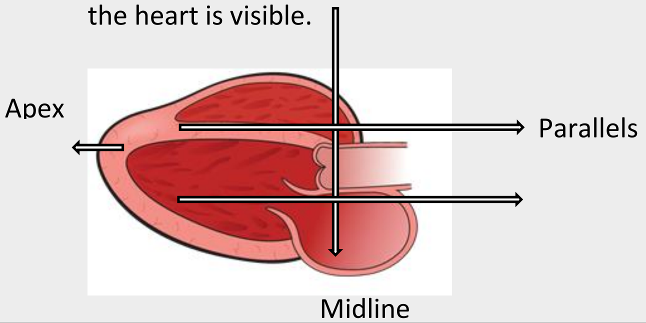 	1. Explain positioning of the patient<br>2. Explain orientation<br>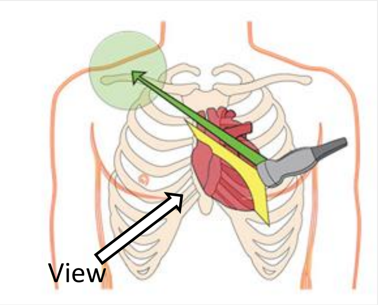<br><br>Source:Philips GmbH Market DACH
☐	**TN 2**	Obtain the parasternal long axis view of the heart, pay attention to adequate image optimization. Orient yourself on the scanning plane and recognize, which side of the heart is located where on the ultrasound image.	1. "Knobology", Explain: *Depth, Gain, Focus, Freeze*<br>*1.* Explain the transducer movements (Tilting, Rocking, Rotating, Sliding)
☐	**TN 3**	Obtain the parasternal long axis view of the heart, pay attention to transducer manipulation and name the anatomical structures.<br>☐ RV, LV<br>☐ LA, RA<br>☐ Ascending + descending aorta<br>☐ Papillary muscles<br>☐ Interventricular septum	Name the anatomical structures (landmarks and quality features of the scan plane)<br>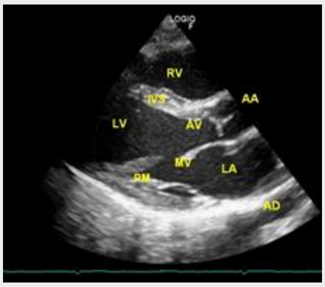
☐	**TN 4**	Obtain the parasternal long axis view of the heart, pay attention to transducer manipulation, image optimization and recognize, which parts of the cardiac wall are demonstrated. Evaluate the different parts of the cardiac wall according to their motion.<br><br>Then rotate the transducer in the parasternal short axis (clockwise 90°), in a way that the marker points to the left shoulder (2 o'clock).<br>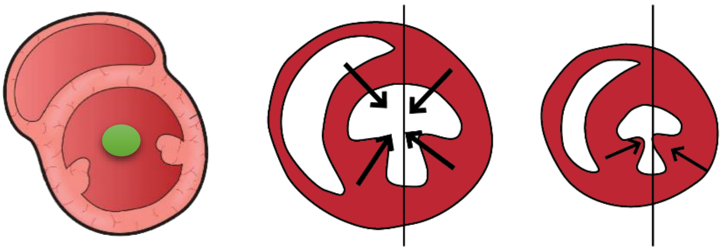<br>Source:Philips GmbH Market DACH	1. Explanation: The plane changes by tilting the transducer cranially or caudally:<br>Level of the aortic valve/- mitral valve/- papillary muscles/- or apex of the heart<br>2. Rotation in the short axis of the heart: Pay attention to image optimization, explain orientation<br>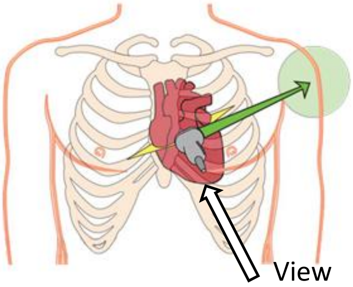<br><br>Source:Philips GmbH Market DACH

J.Weimer; F. Sprengart; A. Dionysopoulou

☑		Scanning guide	Tips for the examiners
☐	TN 1	Starting from the parasternal long axis view, obtain the parasternal short axis view of the heart. Pay attention to image optimization. Orient yourself on the scanning plane and recognize, which side of the heart is located where on the ultrasound image.	Explain the normal systolic function of the heart. The ventricle should be shown in a round form.<br>Tip: Place the mouse pointer in the middle of the ventricle
☐	TN 2	Starting from the parasternal long axis view, obtain the parasternal short axis view of the heart. Pay attention to transducer manipulation, image optimization and name the anatomical structures.	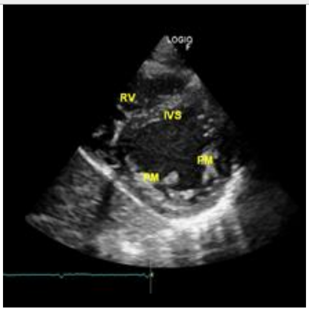
☐	TN 3	Starting from the parasternal long axis view, obtain the parasternal short axis view of the heart. Then make the same in reverse order (short axis to long axis). Use one hand to rotate the transducer while the other hand holds it in place.<br>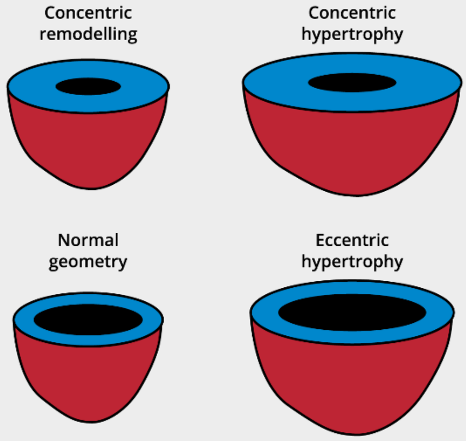	1. Name the anatomical structures (landmarks and quality features of the scan plane)<br>2. Mention the level of the aortic valve, mitral valve and apex of the heart (do not need to be obtained)<br>3. *Pathology: Dilatative Cardiomyopathy*<br>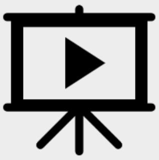
☐	TN 4	Starting from the parasternal long axis view, obtain the parasternal short axis view of the heart. Pay attention to the different parts of the cardiac wall and evaluate the motion of the wall of the left ventricle.<br>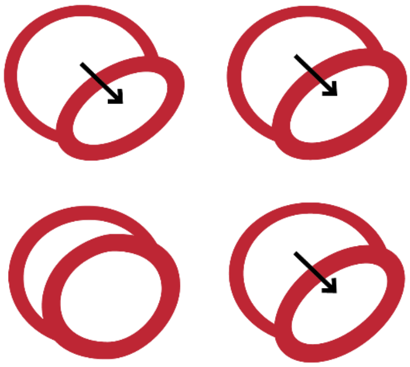	*Pathology: Pulmonary hypertension*<br>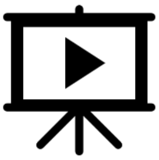

J.Weimer; F. Sprengart; A. Dionysopoulou; J.Weinmann-Menke

Station 2: Apical 4-chamber view + Left flank view + Right flank view

☑		Scanning guide	Tip for examiners
☐	**TN 1**	Obtain the 4-chamber view of the heart. Rotate the transducer in a way so that the marker points to the patient's left side (3 o'clock). Orient yourself on the scanning plane and recognize, which side of the heart is displayed on the image.<br>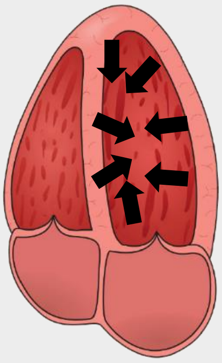	1. Explain positioning of the patient<br>2. Explain: 3 o´clock position, slightly laterally and caudally to the nipple, transducer sliding<br>3. Name the anatomical structures (landmarks and quality features of the scan plane)<br>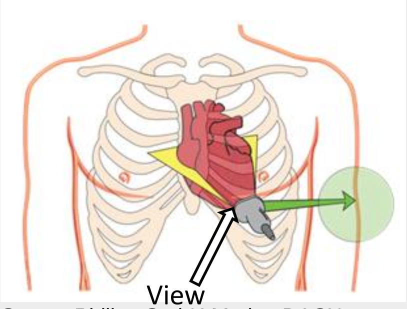<br><br>Source:Philips GmbH Market DACH
☐	**TN 2**	Obtain the parasternal long axis view of the heart, pay attention to transducer manipulation, image optimization and name the anatomical structures (1).<br><br>If possible, demonstrate the origins of the pulmonary veins (2).	1. Once again explain the anatomical structures, ultrasound tips and tricks, transducer manipulation<br>2. Look at how the ventricle contracts during systole (all parts of the wall are pulled towards the middle of the heart)<br>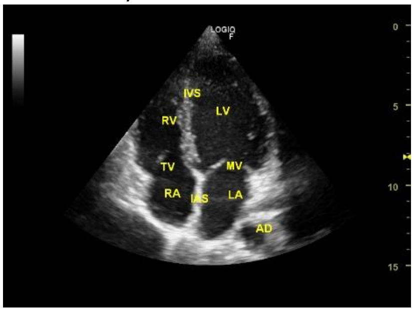
☐	**TN 3**	Obtain the 4-chamber view of the heart and check what effect different movements have on the resulting image. Visually assess the dimensions of each chamber and the global pumping function of the heart.	1. Transducer movements: Rotation, tilting, etc.<br>2. Explanation of the 5-chamber view, here it would be „handbreak downwards“<br>*3. Pathology: Biventricular dysfunction*<br>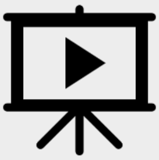
☐	**TN 4**	Obtain the parasternal long axis view of the heart, pay attention to transducer manipulation, image optimization and name the anatomical structures. Focus on the dimensions of each cardiac chamber and then on the left ventricle.	*Pathology: Aneurysm on the apex of the left ventricle*<br>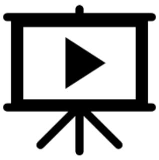

J.Weimer; F. Sprengart; A. Dionysopoulou; J.Weinmann-Menke

☑		Scanning guide	Tip for examiners
☐	**TN 1**	Obtain the right and left flank view, pay attention to transducer manipulation and image optimization.<br>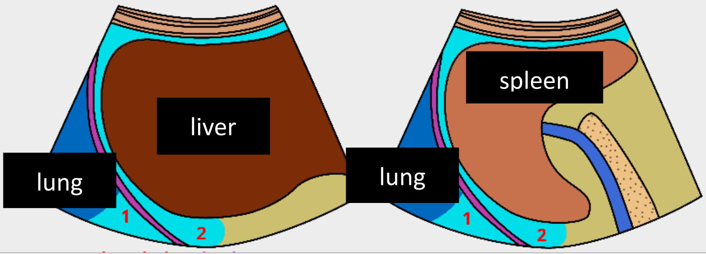 	1. Positioning of the transducer, orientation, image optimization and explanation of the anatomical structures<br>2. Display the other side
☐	**TN 2**	Obtain the right and left flank view, pay attention to transducer manipulation and image optimization, and name the anatomical structures.<br>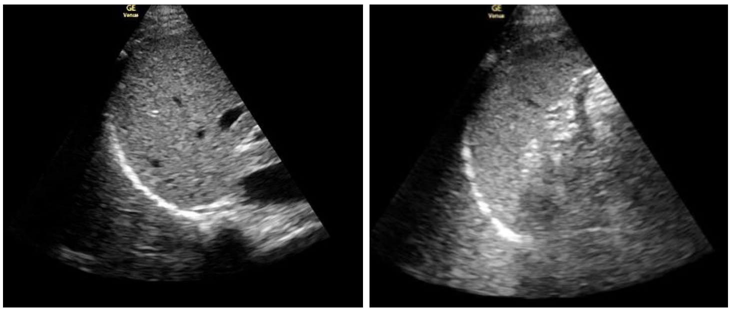	1. Name the anatomical structures
☐	**TN 3**	Obtain the right and left flank view, pay attention to transducer manipulation and image optimization. Show the diaphragm.	*Pathology: Left pleural effusion*<br>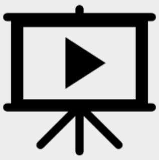
☐	**TN 4**	Obtain the right and left flank view, pay attention to transducer manipulation and image optimization.	*Pathology: Right pleural effusion*<br>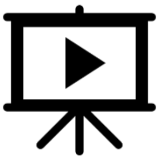

Station 3: Subxiphoid cardiac view + complete examination according to FATE-Protocol

☑		Scanning guide	Tips for the examiners
☐	**TN 1**	Obtain the subxiphoid long axis view of the heart. Position the transducer on the epigastrium as flat as possible and direct it towards the left iliac crest (4-5 o'clock). Pay attention to the 3-finger technique. 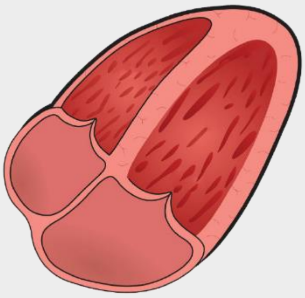	1. Orientation<br>2. Name the anatomical structures (landmarks and quality features of the scan plane)<br>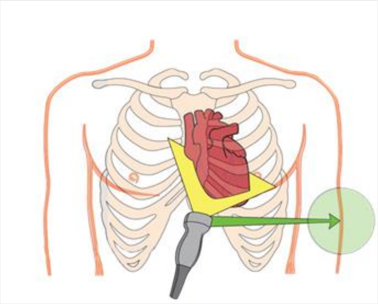<br>Source:Philips GmbH Market DACH
☐	**TN 2**	Obtain the subxiphoid long axis view of the heart, pay attention to transducer manipulation and image optimization. Orient yourself on the scanning plane and recognize, which side of the heart is demonstrated on the image.	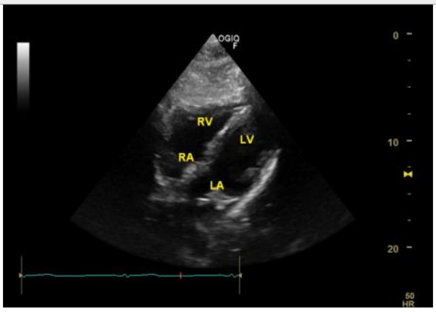
☐	**TN 3**	Obtain the subxiphoid long axis view of the heart, pay attention to transducer manipulation and image optimization and name the anatomical structures. Pay attention to the hyperechoic pericardium and liver. 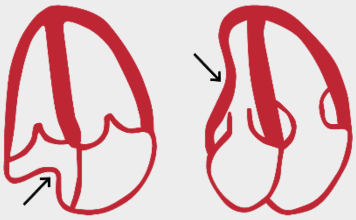	*Pathology: Tamponade*<br>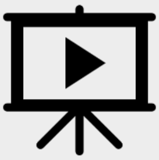
☐	**TN 4**	Obtain the subxiphoid long axis view of the heart, pay attention to transducer manipulation and image optimization and name the anatomical structures.	*Pathology: Myxoma*<br>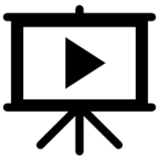
☐	**TN 1**	Carry out a complete examination according to the FATE protocol. Pay attention to the correct order of the scanning planes, the position of the transducer and image optimization.	*Pathology: Anterior myocardial infarction with pericardial effusion*<br>1. Show where the infraction is located (ant./inf./septal., etc.)

J.Weimer; F. Sprengart; A. Dionysopoulou; J.Weinmann-Menke

☑		Scanning guide	Tips for the examiners
☐	**TN 2**	Carry out a complete examination according to the FATE protocol. Pay attention to the correct order of the scanning planes, the position of the transducer and image optimization.	*Pathology: Pulmonary hypertension*
☐	**TN 3**	Carry out a complete examination according to the FATE protocol. Pay attention to the correct order of the scanning planes, the position of the transducer and image optimization.	*Pathology: Acute lateral myocardial infraction* 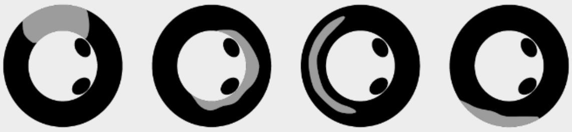
☐	**TN 4**	Carry out a complete examination according to the FATE protocol. Pay attention to the correct order of the scanning planes, the position of the transducer and image optimization.	*Pathology: Dilatative cardiomyopathy*
