# Supplementary material for: Simulator training in focus assessed transthoracic echocardiography (FATE) for undergraduate medical students: results from the FateSim randomized controlled trial
Source: BMC Med Educ. 2025 Jan 4;25:21. doi: 10.1186/s12909-024-06564-y (PMC11699650; doi:10.1186/s12909-024-06564-y)
Supplement: Supplementary file 3 — Supplementary Material 3 [file 12909_2024_6564_MOESM3_ESM.pdf]

# Station 1: Introduction to the devices + parasternal long axis view + parasternal short axis view

| ☑ | | Scanning guide | Tip for the examiners |
|---|---|---|---|
| ☐ | TN 1 | Obtain the parasternal long axis view of the heart | *Normal patient*<br>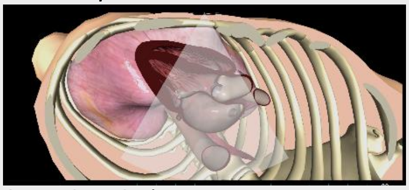<br>Soure:CAE GmbH<br>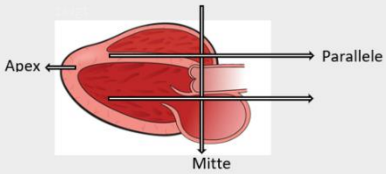<br><br>Source:Philips GmbH Market DACH<br>1. *Layout ➪ AR Only*<br>2. After the image has been acquired: *Beam ➪ guide* (Describe image orientation)<br>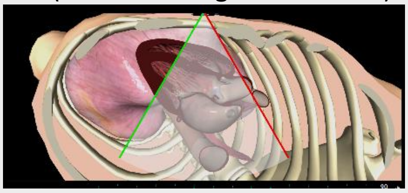<br>Soure:CAE GmbH<br>3. After the image has been acquired: *Layout ➪ split view*<br>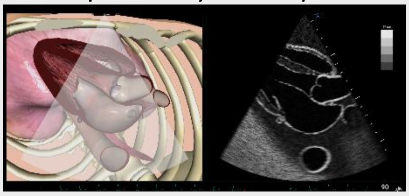<br>Soure:CAE GmbH |
| ☐ | TN 2 | Obtain the parasternal long axis view of the heart, pay attention to adequate image optimization | *Normal patient,* name the buttons on the device<br>*Depth*<br>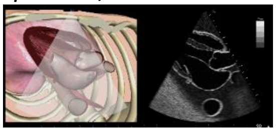<br>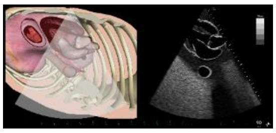<br>*Gain*<br>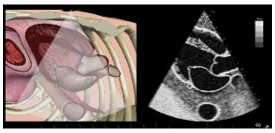<br>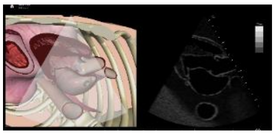<br>Soure:CAE GmbH<br>*Beam wide*<br>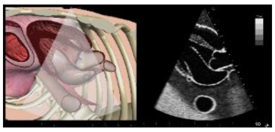<br>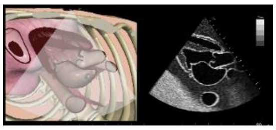 |
| | TN 3 | Obtain the parasternal long axis view of the heart, pay attention to transducer manipulation and name the anatomical structures | *Normal patient*<br>1. *Beam ➪ Ultrasound*<br>Name the anatomical structures (landmarks and quality features)<br>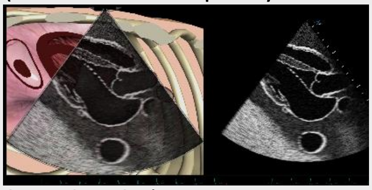<br>Soure:CAE GmbH<br>2. *Beam ➪ Guide* to explanation of probe orientation<br>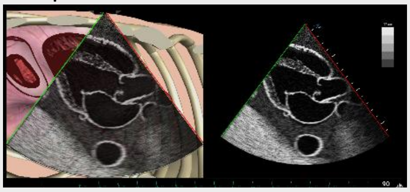<br>Soure:CAE GmbH |
| ☐ | TN 4 | Obtain the parasternal long axis view of the heart, pay attention to transducer manipulation, image optimization and name the anatomical structures (1)<br><br>Rotate the transducer to the parasternal short axis view and pay attention to image optimization (2) | *Pathology: dilated cardiomyopathy – very severe left ventricular systolic dysfunction*<br>1. Name the pathologic findings, noncontractile left ventricle, systolic dysfunction<br>2. Rotate the transducer to the short axis view of the heart: pay attention to image optimization *Beam ➪ Guide* to orientation explanation |

J.Weimer; F. Sprengart; A. Dionysopoulou, CAE Healthcare

| ☑ | | Scanning guide | Tip for the examiners |
|---|---|---|---|
| | | Describe the pathologic findings (3) | 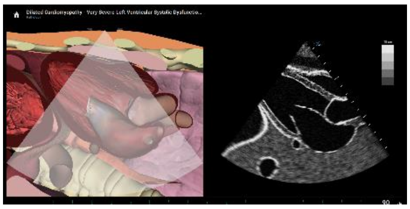<br>Soure:CAE GmbH<br>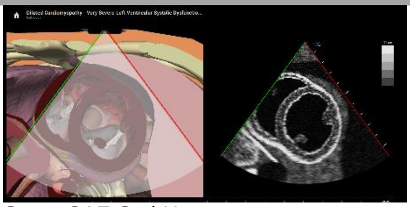<br>Soure:CAE GmbH<br>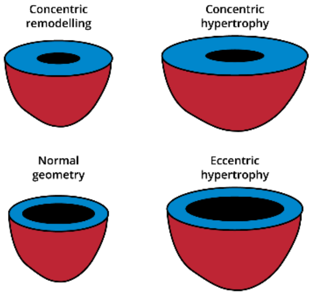<br><br>3. Describe the pathologic findings: Systolic dysfunction at the level of the papillary muscles (eye balling), the ventricle contracts barely.<br>Tip: Place the mouse pointer in the middle of the ventricle |
| ☐ | **TN 1** | Starting from the parasternal long axis view , obtain the parasternal short axis view of the heart<br>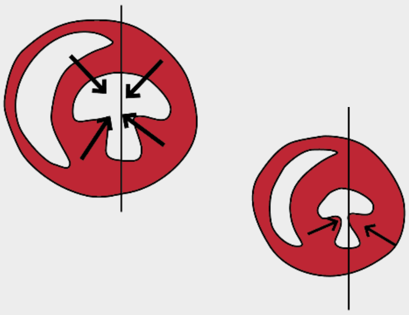 | *Normal patient*<br>1. Explain the normal systolic function<br>Tip: Place the mouse pointer in the middle of the ventricle<br>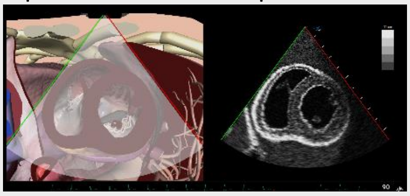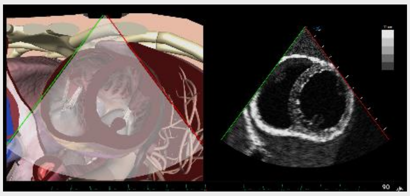<br>Soure:CAE GmbH<br>*2. Beam ➩ Ultrasound*<br>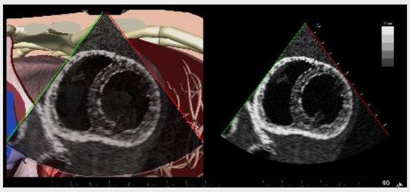<br>Soure:CAE GmbH<br>*3.* Explain the transducer movements (Tilting, Rocking, Rotating, Sliding) |
| ☐ | **TN 2** | Starting from the parasternal long axis view, obtain the parasternal short axis view of the heart. Pay attention to transducer manipulation, image optimization and name the anatomical structures | *Normal patient*<br>1. Remain on the same scan plane as before<br>2. After obtaining the scan plane, change to *Layout ➩ AR Only + Beam ➩ Transparent*<br>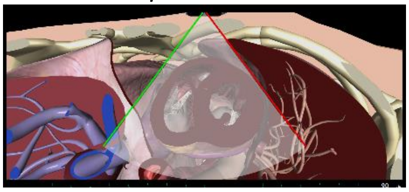<br>Soure:CAE GmbH |
| ☐ | **TN 3** | Starting from the parasternal long axis view , obtain the parasternal short axis view of the heart (AR only) (1)<br><br>Name the anatomical structures (large US) (2) | *Normal patient*<br>1. Remain on AR only<br>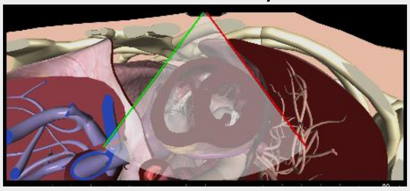<br>Soure:CAE GmbH<br>2. After obtaining the scan plane correctly: *Layout ➩ Large US*<br>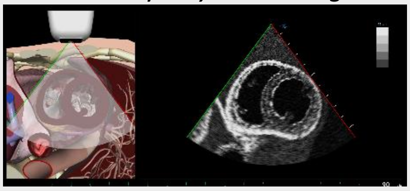<br>Soure:CAE GmbH |

| ☑ | | Scanning guide | Tip for the examiners |
|---|---|---|---|
| | | | 3. Mention the level of the aortic valve, mitral valve and apex of the heart (do not need to be obtained) |
| ☐ | **TN 4** | Starting from the parasternal long axis view , obtain the parasternal short axis view of the heart (1)<br><br>Describe the pathologic findings (2) | *Pathology: pulmonary hypertension*<br>1. Description of the D-Sign in right ventricular strain, the interventricular septum is pressed towards the left ventricle<br>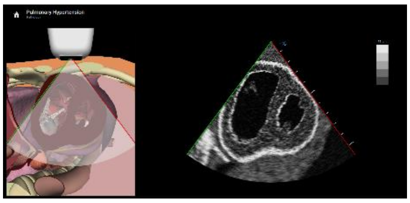<br>Soure:CAE GmbH<br>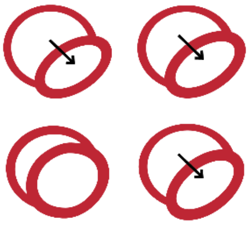 |

## Station 2: Apical 4-chamber view + Left flank view + Right flank view

| ☑ | | Scanning guide | Tip for the examiners |
|---|---|---|---|
| ☐ | **TN 1** | Obtain the apical 4-chamber view of the heart<br>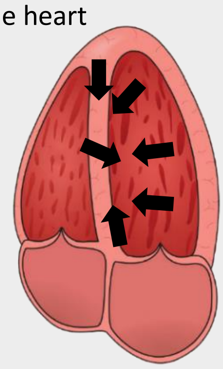<br>Source:Philips GmbH Market DACH | *Normal patient*<br>*1. Layout ➩ AR only*<br>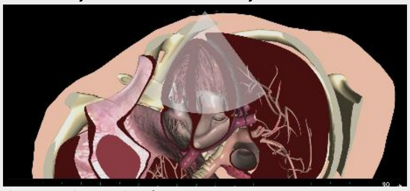<br>Soure:CAE GmbH<br>2. Explain: Position at 3 o´clock, sliding of the transducer, quality features of the scan plane<br>3. After obtaining the scan plane correctly: *Layout ➩ split view*<br>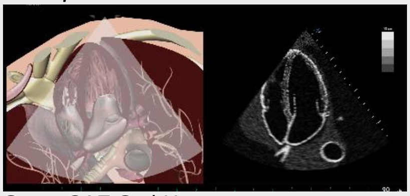<br>Soure:CAE GmbH |
| ☐ | **TN 2** | Obtain the apical 4-chamber view of the heart, pay attention to transducer manipulation, image optimization and name the anatomical structures (1)<br><br>Show the origins of the pulmonary veins (2) | *Normal patient*<br>*1. Layout ➩ split view*<br>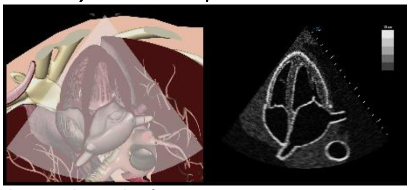<br>Soure:CAE GmbH<br>2. Once again explain the anatomical structures, ultrasound tips and tricks, transducer manipulation<br>3. Watch how the ventricle contracts during systole (all parts of the wall are pulled towards the middle of the heart) |
| ☐ | **TN 3** | Obtain the apical 4-chamber view of the heart (1)<br><br>Describe the pathologic findings (2) | *Pathology: severe biventricular dysfunction, case 10*<br>1. Pay attention to the wall of the heart, show the global systolic dysfunction<br>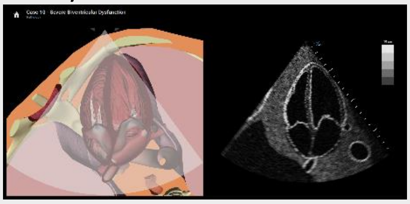<br>Soure:CAE GmbH |

| ☑ | | Scanning guide | Tip for the examiners |
|---|---|---|---|
| ☐ | **TN 4** | Obtain the apical 4-chamber view of the heart, pay attention to transducer manipulation, image optimization and name the anatomical structures (1)<br><br>Describe the pathologic findings (2) | *Pathology: left ventricular apical aneurysm with thrombus*<br>1. Place the transducer as caudally as possible, in order to identify the thrombus<br>2. Explain the pathologic findings: No contraction seen at the apex of the heart, contraction can be seen every now and then towards the mitral valve<br>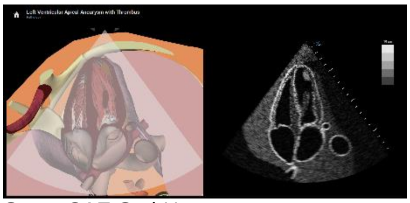<br>Soure:CAE GmbH |
| ☐ | **TN 1** | Obtain the right and left flank view, pay attention to transducer manipulation and image optimization | *Normal patient*<br>1. *Layout ➩Obtain an image of the lungs* (in case it has been switched off previously)<br>2. *Layout ➩ split view*<br>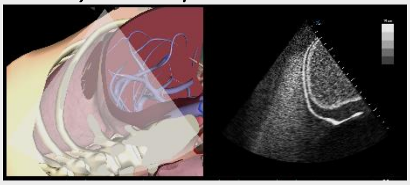<br>Soure:CAE GmbH<br>3. Image optimization and explanation of the anatomical structures<br>4. Display the other side: *Beam ➩ Guide* to orientation cranial/caudal<br>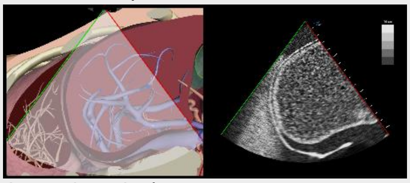<br>Soure:CAE GmbH |
| ☐ | **TN 2** | Obtain the right and left flank view, pay attention to transducer manipulation and image optimization and explain the anatomical structures | *Normal patient*<br>1. Left flank *Beam ➩ Ultrasound*<br>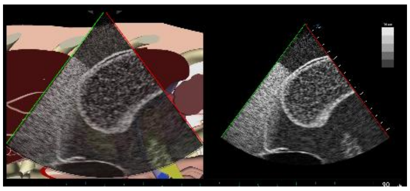<br>Soure:CAE GmbH<br>2. Explain the anatomical structures |
| ☐ | **TN 3** | Obtain the right (1) and left (2) flank view, pay attention to transducer manipulation and image optimization.<br><br>Describe the pathologic findings (3) | *Pathology: left pleural effusion case 09*<br>1. Explain the pathogenesis, hypoechoic effusion<br>2. Ultrasound tip: Rotate the transducer between the ribs (scan through the intercostal space), move ventrally<br>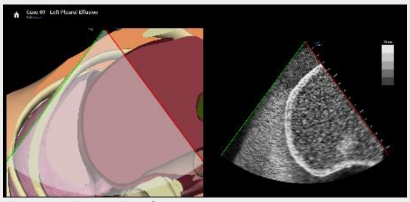 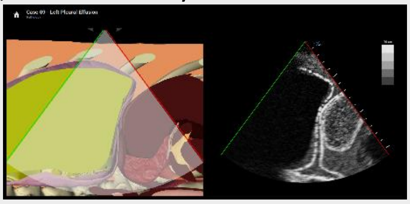<br>Soure:CAE GmbH |
| ☐ | **TN 4** | Obtain the right (1) and left (2) flank view, pay attention to transducer manipulation and image optimization.<br><br>Describe the pathologic findings (3) | *Pathology: right pleural effusion*<br>1. See instructions above<br>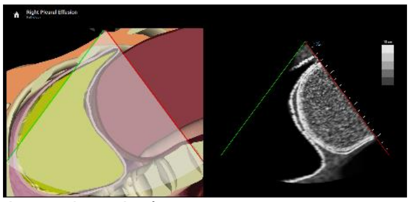 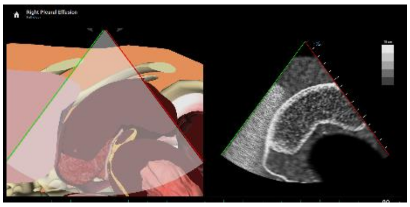<br>Soure:CAE GmbH |

# Station 3: Subxiphoid cardiac view + complete examination according to FATE-Protocol

| ☑ | | Scanning guide | Tip for the examiners |
|---|---|---|---|
| ☐ | **TN 1** | Obtain the subxiphoid long axis view of the heart | *Normal patient*<br>Pay attention to coupling! Hold the transducer at the base (3 finger grip)<br>1. *Layout* ➩ *split view*<br>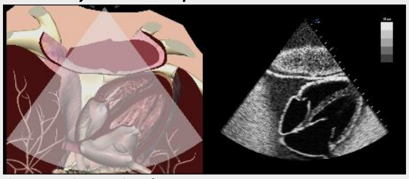<br>Soure:CAE GmbH<br>2. After obtaining the scan plane correctly: *Beam* ➩ *Guide* zur Orientation<br>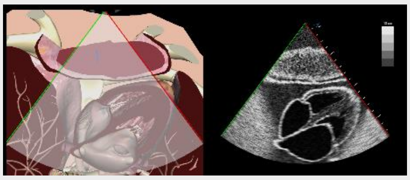<br>Soure:CAE GmbH<br>3. Explain the anatomical structures (landmarks and quality features of the scan plane) |
| ☐ | **TN 2** | Obtain the subxiphoid long axis view of the heart, pay attention to transducer manipulation and image optimization and name the anatomical structures | *Normal patient*<br>1. Remain on the previous scan plane<br>2. After obtaining the scan plane correctly: *Layout* ➩ *AR only*<br>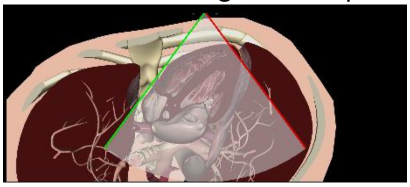<br>Soure:CAE GmbH |
| ☐ | **TN 3** | Obtain the subxiphoid long axis view of the heart, pay attention to transducer manipulation and image optimization and name the anatomical structures (1)<br><br>Describe the pathologic findings (2) | *Pathology: tamponade*<br>1. Layout ➩ Large US<br>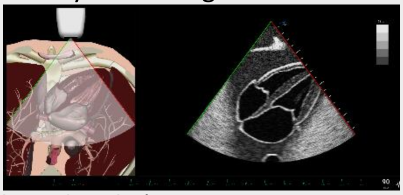<br>Soure:CAE GmbH<br>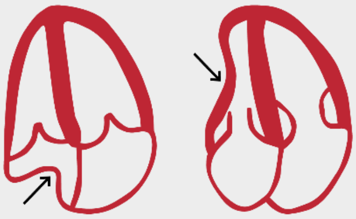<br>2. Explain the pathologic findings (hypoechoic fluid, Swinging heart due to lack of space)<br>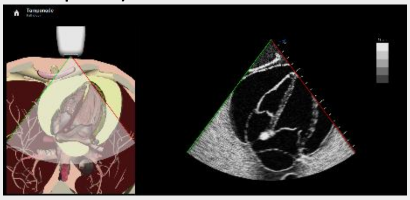<br>Soure:CAE GmbH<br>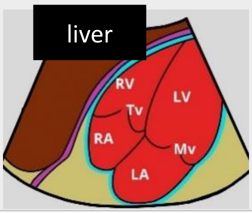<br> |
| ☐ | **TN 4** | Obtain the subxiphoid long axis view of the heart, pay attention to transducer manipulation and image optimization and name the anatomical structures (1)<br><br>Describe the pathologic findings (2) | *Pathology: myxoma*<br>1. Explain the pathologic findings<br>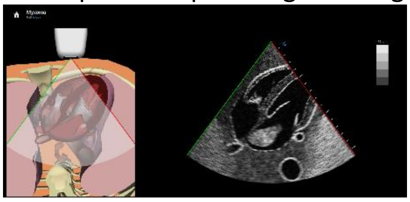<br>Soure:CAE GmbH |

| ☑ | | Scanning guide | Tip for the examiners |
|---|---|---|---|
| ☐ | **TN 1** | Carry out a complete examination according to the FATE protocol. Pay attention to the correct order of the scanning planes, the position of the transducer and image optimization (1)<br><br>Describe the pathologic findings (2) | *Pathology: recent anterior myocardial infarction with pericardial effusion*<br>2. Explain the pathologic findings<br>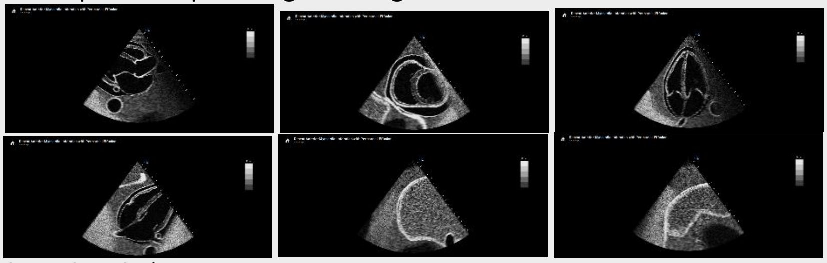<br>Soure:CAE GmbH |
| ☐ | **TN 2** | Carry out a complete examination according to the FATE protocol. Pay attention to the correct order of the scanning planes, the position of the transducer and image optimization (1)<br><br>Describe the pathologic findings (2) | *Pathology: pulmonary hypertension*<br>2. Explain the pathologic findings (Description of the D-Sign in right ventricular strain, the interventricular septum is pressed towards the left ventricle)<br>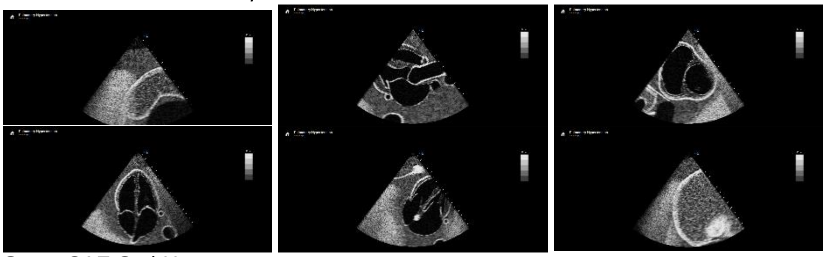<br>Soure:CAE GmbH |
| ☐ | **TN 3** | Carry out a complete examination according to the FATE protocol. Pay attention to the correct order of the scanning planes, the position of the transducer and image optimization (1)<br><br>Describe the pathologic findings (2) | *Pathology: acute lateral myocardial infarction*<br>1. Explain the pathologic findings (the wall does not move)<br>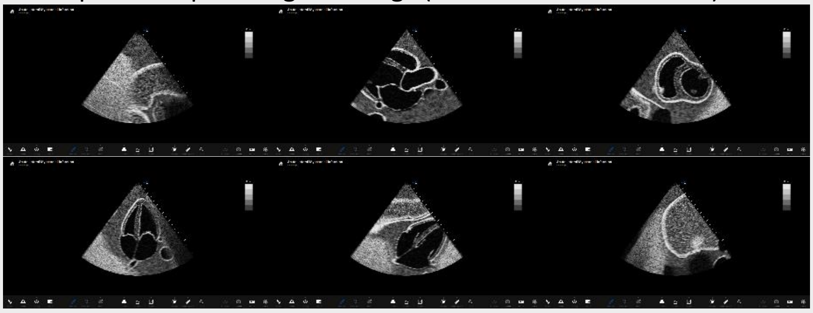<br>Soure:CAE GmbH<br>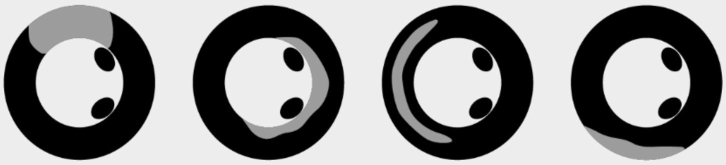 |
| ☐ | **TN 4** | Carry out a complete examination according to the FATE protocol. Pay attention to the correct order of the scanning planes, the position of the transducer and image optimization (1)<br><br>Describe the pathologic findings (2) | *Pathology: dilatated cardiomyopathy – very severe left ventricular systolic dysfunction*<br>(Attention: If one lung is collapsed, you get the impression that there is a pleural effusion)<br>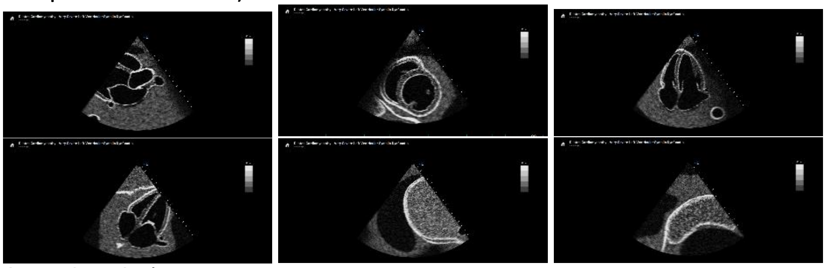<br>Soure:CAE GmbH |
