# Supplementary material for: Simulator training in focus assessed transthoracic echocardiography (FATE) for undergraduate medical students: results from the FateSim randomized controlled trial
Source: BMC Med Educ. 2025 Jan 4;25:21. doi: 10.1186/s12909-024-06564-y (PMC11699650; doi:10.1186/s12909-024-06564-y)
Supplement: Supplementary file 4 — Supplementary Material 4 [file 12909_2024_6564_MOESM4_ESM.pdf]

VIMEDIX PATHOLOGIES

Different modules and packages offer pathologies that focus on specific cases during ultrasound training. All of the following modules and packages are available for purchase.

Transthoracic Echocardiography (TTE) Module

The transthoracic echocardiography (TTE) module contains the following pathologies:

Dilated Cardiomyopathy – Severe Biventricular Systolic Dysfunction

Hyperdynamic Left Ventricular Systolic Function

Normal

Normal, Patient 2 (Montreal Heart Institute heart template, enhanced lung)

Normal, Patient 3 (Montreal Heart Institute heart template, enhanced lung, enhanced abdomen)

Recent Anterior Myocardial Infarction with Pericardial Effusion

Transesophageal Echocardiography (TEE) Module

The transesophageal echocardiography (TEE) module contains the following pathologies:

Dilated Cardiomyopathy – Severe Biventricular Systolic Dysfunction

Hyperdynamic Left Ventricular Systolic Function

Normal

Normal, Patient 2 (Montreal Heart Institute heart template, enhanced lung)

Normal, Patient 3 (Montreal Heart Institute heart template, enhanced lung, enhanced abdomen)

Recent Anterior Myocardial Infarction with Pericardial Effusion

Additional Cardiac Pathology Packages

The following packages are sold separately from the two cardiac module options.

Cardiac Package 1

Anterior Myocardial Infarction in a COPD Patient

Biologic Prosthetic Valve in Aortic Position

Dilated Cardiomyopathy – Mild Left Ventricular Systolic Dysfunction

Dilated Cardiomyopathy – Very Severe Left Ventricular Systolic Dysfunction in a COPD Patient

Left Pleural Effusion

Left Ventricular Apical Aneurysm with Thrombus

Mechanical Prosthetic Valve (Bileaflet) in Aortic and Mitral Position

Mechanical Prosthetic Valve (Bileaflet) in Mitral Position

Normal Heart in a COPD Patient

Tamponade

Cardiac Package 2

Acute Anterior Myocardial Infarction

Acute Lateral Myocardial Infarction in a COPD Patient

Aortic Valve Infective Endocarditis

Asystole

Coarse Ventricular Fibrillation

Dilated Cardiomyopathy – Very Severe Left Ventricular Systolic Dysfunction

Dilated Cardiomyopathy – Mild Left Ventricular Systolic Dysfunction in a COPD Patient

Fine Ventricular Fibrillation

Pulmonary Hypertension

Pulmonary Hypertension in a COPD Patient

Cardiac Package 3

Acute Inferior Myocardial Infarction

Acute Lateral Myocardial Infarction

Acute Right Ventricular Myocardial Infarction

Aortic Dissection – Type B

Aortic Stenosis - Valvular

Billowing Mitral valve – two leaflets

Bicuspid Aortic Valve

Dilated Cardiomyopathy – Severe Left Ventricular Systolic Dysfunction

Myxoma

Right Pleural Effusion

Cardiac Package 4

Acute Inferior and Right Ventricular Myocardial Infarction with Ventricular Septal Defect

Acute Inferior Myocardial Infarction with Right Ventricular Myocardial Infarction

Aortic Insufficiency

Atrial Septal Defect - small

Billowing Mitral Valve

Cardiac Arrest Standstill in a COPD patient

Coronary Artery Disease – Wall Motion Abnormalities in the 3 Coronary Territories

Dilated Cardiomyopathy – Moderate Biventricular Systolic Dysfunction

Left Atrial Appendage Thrombus

Thrombus in Transit Patent Foramen Ovale

Cardiac Mix & Match Package 1

This Cardiac Mix & Match package includes pathologies from all four of the cardiac packages and is tailored to the needs of point-of-care healthcare professionals.

Acute Lateral Myocardial Infarction

Anterior Myocardial Infarction in a COPD Patient

Aortic Stenosis – valvular

Dilated Cardiomyopathy – Moderate Biventricular Systolic Dysfunction

Fine Ventricular Fibrillation

Mechanical Prosthetic Valve (bileaflet) in Aortic and Mitral Position

Myxoma

Pulmonary Hypertension

Right Pleural Effusion

Tamponade

Montreal Heart Institute

This Montreal Heart Institute package includes pathologies created in collaboration with the Montreal Heart Institute.

Amyloidosis

CMP - Dilated

CMP - Hypertrophic

Ebstein's Anomaly - ASD

LV Apical Thrombus

Mitral Valve Prolapse

Mitral Valve - Rheumatic Disease

Myxoma

Takotsubo

VSD (CIV) Post-Infarct

Montreal Heart Institute (MHI) Acute Complex Pathology+ Package

This package was developed in collaboration with the Montreal Heart Institute (MHI), using our enhanced heart and lung anatomical template, which includes animated lungs and IVC.

Abdominal Compartment Syndrome

Dynamic Right Ventricular Outflow Tract Obstruction

Floating Pulmonary Embolism

Full Stomach

Inferior Vena Cava Stenosis

Isolated Left Atrial Tamponade

Isolated Right Atrial Tamponade

Left Ventricular Outflow Tract Obstruction

Mechanical Right Ventricular Outflow Tract Obstruction

Reduced Mean Systemic Pressure (Reduced Preload) From Liver Abscess

Reduced Mean Systemic Pressure (Respiratory Variation Of Superior Vena Cava)

Right Pneumothorax And Right Heart Collapse

Right-sided Carbon Dioxide Or Air Embolism

Emergency Ultrasound Pathology Package

The Emergency Ultrasound Pathology Package contains emergency medical conditions requiring rapid diagnosis at the point of care and includes the following pathologies:

Right Atrium Tamponade

Severe Hypovolemia

LVOT Obstruction LVH Post AVR

Acute RV Failure

Air Embolism

Extensive Myocardial Ischemia

Aortic Dissection

Large Cardiac Mass

Acute MR Post AVR

Prosthesis Dysfunction Post MVR

Abdominal Base Module

The Abdominal Base module contains the following pathologies:

Normal

Normal, Patient 2

Normal, Patient 3

Hydatid Cyst of the Liver

Liver Hepatocellular Carcinoma Hypoechoic

Multilocular Intra-Abdominal Abscess

Free Fluid - Hepato-Renal Reflection (Morrison's Pouch - Small)

Free Fluid - Retro-Vesical Reflection (Large)

Free Fluid – Spleno-Renal Reflection (Medium)

Additional Abdominal Pathology Packages

The following packages are sold separately from the two abdominal module options.

Abdominal Package 1

Acute Cholecystitis

Angiomyolipoma

Bacterian Hepatic Abscess

Cholecystitis with Gallstone

Choledocolithiasis

Exophytic Renal Cyst

Hepatomegaly

Kidney Stones

Pancreatic Pseudocyst

Splenomegaly

Abdominal Package 2

- Chronic Pancreatitis
- Gallbladder Polyp
- Hepatic Haemangioma
- Hepatic Heterogeneous Metastases
- Hepatic Homogeneous Metastases
- Hepatic Steatosis
- Large Gallbladder Polyp
- Renal Cyst
- Splenic Cyst
- Splenic Haemangioma

Abdominal Package 3

- Bochdalek Hernia - Left Side
- Cholelithiasis
- Gallstones
- Hepatic Cirrhosis with Portal Hypertension
- Liver Hepatoma
- Heterogeneous Lesion
- Pancreatitis
- Pyelonephritis
- Renal Abscess
- Renal Cyst Rupture
- Schlerosing Cholangitis

Abdominal Package 4

- Adenocarcinoma of the Gallbladder
- Bochdalek Hernia
- Calcified Granulomas
- Focal Nodular Hyperplasia
- Hepatic Cirrhosis
- Hepatic Cirrhosis with Ascites
- Left Hydronephrosis
- Liver Adenoma
- Liver Hepatocellular Carcinoma Hyperechoic
- Liver Hepatoma Hyperechoic Lesion (Large)

Abdominal Aortic Aneurysm (AAA) Pathology+ Package

AAA - Medium Suprarenal

AAA - Small Renal

AAA - Medium Renal

Left Iliac Artery Aneurysm

Each AAA pathology has the following modality options that can be turned on and off:

- Digestive system gas
- Dissection
- Free fluid
- Mural Thrombus
- Pericardial Fluid

FAST Package 1

FAST Package 1 contains 10 cases involving various levels and combinations of free fluid around the heart, lungs and abdomen consistent with traumatic injuries.

The following pathologies are included in the FAST Package 1:

Free Fluid - Retro-Vesical Reflection (Small)

Free Fluid - Retro-Vesical Reflection (Medium)

Free Fluid - Splenal-Renal Reflection (Small)

Free Fluid - Supra-Splenal

Free Fluid - Above the Spleen with Hemothorax

Free Fluid - Bilateral Renal Reflection (Small)

Free Fluid - Hepato-Renal Reflection (Morrison's Pouch - Large)

Left Lateral Trauma

Spleen Rupture

Small Pericardial Effusion

FAST Mix & Match Package

This FAST Mix & Match package includes selected pathologies from all of the cardiac and abdominal packages and is tailored to the needs of point-of-care healthcare professionals.

Abdominal Aortic Aneurysm

Abdominal Aortic Dissection

Aortic Dissection – Type B

Free Fluid - Hepato-Renal Reflection (Morrison's Pouch – medium)

Free Fluid - Retro-Vesicular Reflection (Medium)

Free Fluid - Splenal-Renal Reflection (Large)

Free Fluid - Splenal-Renal Reflection (Small)

Left Pleural Effusion

Right Pleural Effusion

Tamponade

Pleural Pathology Package

The Pleural Pathology Package provides lung pathologies featuring fully animated lung respiration and respiratory variation of the inferior vena cava (IVC). The following cases are part of the Pleural Pathology Package:

Bilateral Diaphragmatic Dysfunction

Bilateral Pulmonary Edema

Central Pneumonia

Complete Pleural Effusion

Empyema

Normal, Enhanced Heart and Lungs

Pneumonia

Pneumothorax

Small Pleural Effusion

Unilateral Diaphragmatic Dysfunction
