# Supplementary material for: Simulator training in focus assessed transthoracic echocardiography (FATE) for undergraduate medical students: results from the FateSim randomized controlled trial
Source: BMC Med Educ. 2025 Jan 4;25:21. doi: 10.1186/s12909-024-06564-y (PMC11699650; doi:10.1186/s12909-024-06564-y)
Supplement: Supplementary file 5 — Supplementary Material 5 [file 12909_2024_6564_MOESM5_ESM.pdf]

Question 1

- Complete the following sentence: The correct name for "dark" areas on an ultrasound image is:

  ____

- Complete the following sentence: The correct name for "bright" areas on an ultrasound image is:

  ____

Question 2

Which **transducer** is shown here?

Transducer:

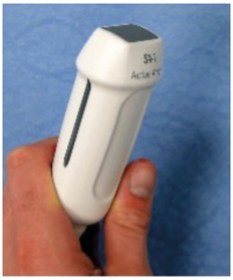

Question 3

Which **sectional scan planes** are shown here? Label them on the images:

1. Sectional scan plane:
2. Sectional scan plane:
3. Sectional scan plane:

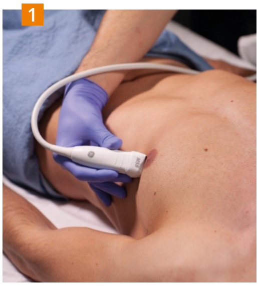

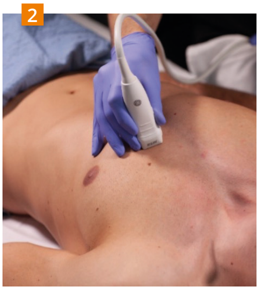

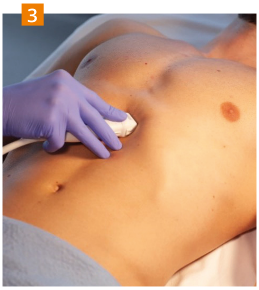

Question 4

Which **ultrasound image results** from the **scanning approach** seen here?

1: ☐ 2: ☐ 3: ☐ 4: ☐ 5: ☐

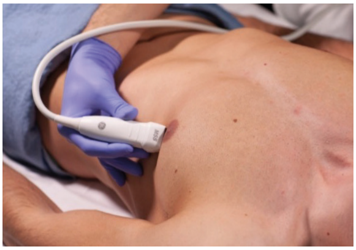

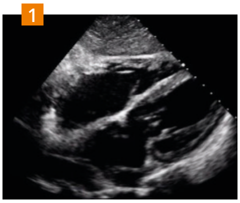

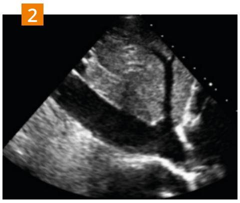

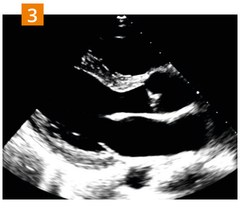

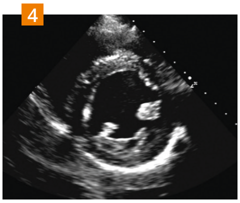

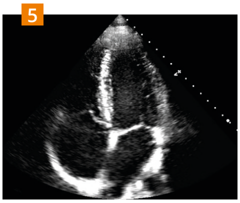

Question 5

Which **image** on the **ultrasound simulator results** from the **scanning approach** seen here?

1: ☐ 2: ☐ 3: ☐ 4: ☐ 5: ☐

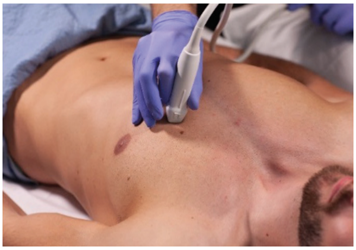

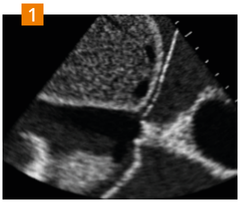

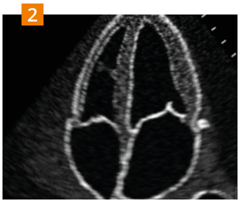

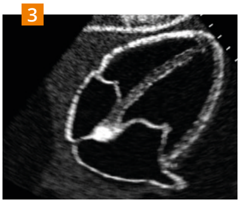

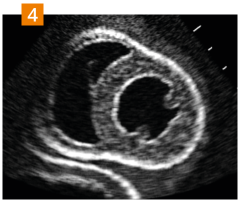

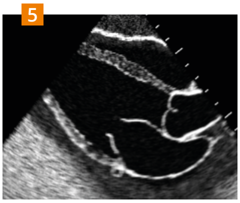

Question 6

Through which **scanning approach** is the **ultrasound image**, seen here, obtained?

1: ☐ 2: ☐ 3: ☐ 4: ☐ 5: ☐

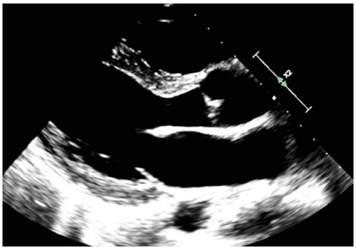

1
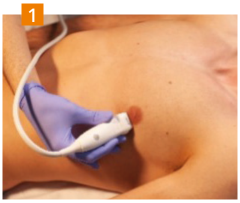

2
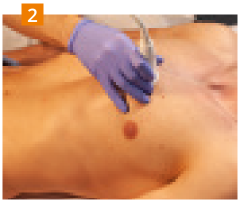

3
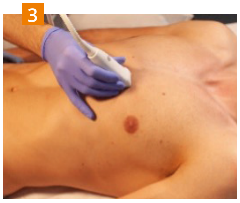

4
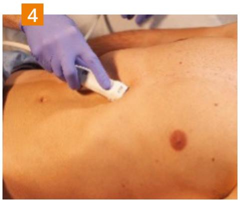

5
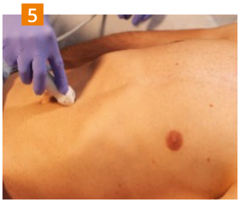

Question 7

Through which **scanning approach** is the **image** on the **ultrasound simulator**, seen here, obtained?

1: ☐ 2: ☐ 3: ☐ 4: ☐ 5: ☐

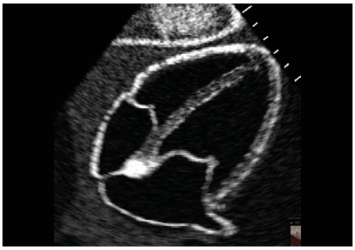

1
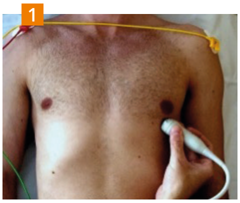

2
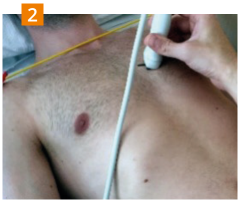

3
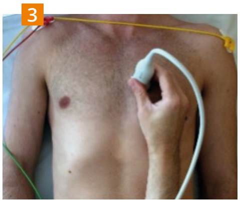

4
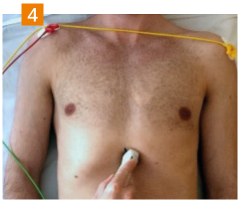

5
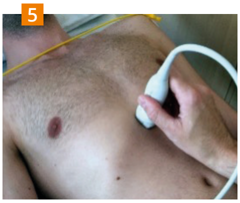

Question 8

Which **structures** are marked on the image? Describe exactly.

A
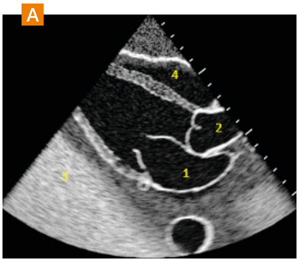

B
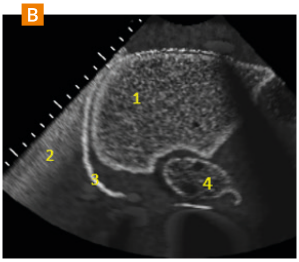

C
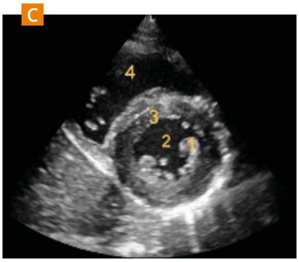

A1: A2:
A3: A4:

B1: B2:
B3: B4:

C1: C2:
C3: C4:

Question 9

Which **pathologies** are marked on the image? Describe exactly.

1
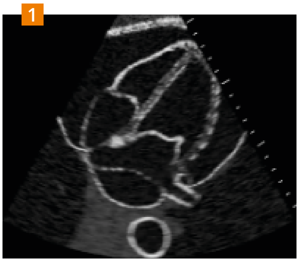

2
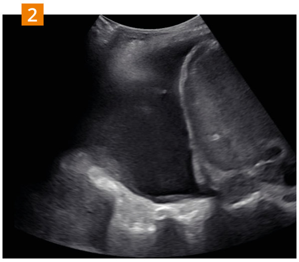

3
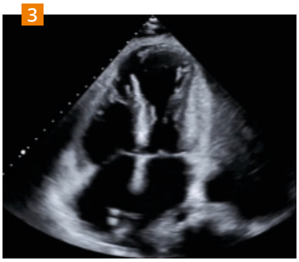

1. Pathology:

2. Pathology:

3. Pathology:
