# Supplementary material for: Simulator training in focus assessed transthoracic echocardiography (FATE) for undergraduate medical students: results from the FateSim randomized controlled trial
Source: BMC Med Educ. 2025 Jan 4;25:21. doi: 10.1186/s12909-024-06564-y (PMC11699650; doi:10.1186/s12909-024-06564-y)
Supplement: Supplementary file 6 — Supplementary Material 6 [file 12909_2024_6564_MOESM6_ESM.pdf]

FATE-Protocol: Case

Case: You are a resident doctor in the emergency department of the university hospital. A 72-year old patient presents with shortness of breath and mild chest pain. You initially perform an ECG. For further assessment, you would like to carry out a focused cardiac ultrasound examination.

Task 1: Guide the patient through the ultrasound examination **(please ask the participants on the simulator specifically about this)**

Task 2 + 3: Correctly obtain the standard scan planes of the FATE protocol (including quality features, max. 90 seconds per plane), in order to get an overview of the heart function and possible underlying pathologies.
Freeze **each image**, **show and name the key anatomical structures**. Please do not take any measurements.

Tips for the examiners:

- **Time: 8 minutes.** Should the participants not manage to obtain the scan planes themselves, please provide assistance, in order for them to be able to perform Task 3 (alternatively, show pictures on the ultrasound machine/simulator). **Do not spend more than 90 seconds per scan plane!**
- Assess ultrasound scanning technique during the examination process at FATE 1 and again at the end: 6 evaluation criteria (orientation, positioning of the transducer, coupling, transducer manipulation, image optimization and operation of the device (ultrasound machine/simulator). 2 points per criterion (independent and confident), 1 point if verbal instructions are necessary, 0 points if assistance is required. Please make sure that your left hand is on the ultrasound machine/simulator **(Adjust the device before starting!!).**
- Attribute 2 points for issuing **breathing commands** (i.e. inhale, exhale, hold breath) and **positioning of the patient,** provided, that no instructions were given and the scan plane could be obtained correctly.
- Please add up the **points** only after the exam and **do not give the evaluation form away!**

The **evaluation form** contains three possible options for each task:

- **Green:** The examinee carries out the task completely correctly/optimally.
- **Yellow:** The examinee completes the task with some difficulty/with assistance from the examiner.
- **Red:** The examinee was unable to complete the tasks adequately, despite assistance from the examiner.

Test objectives:

- The examinee is proficient in handling the transducer and adjusting the image in relation to:
  - Orientation
  - Positioning of the transducer
  - Coupling
  - Image magnification and image optimization
- The examinee is able to guide the patient through the examination.
- The examinee can carry out the examination according to the guidelines with regard to:
- … obtaining **the scan planes of the FATE protocol**
- … **naming the key anatomical structures on each scan plane**
- … **explaining the images** on each plane respectively
- … **the overall performance**

J.Weimer; F. Sprengart; A. Dionysopoulou, J.Weinmann-Menke

Task sheet FATE-Protocol DOPS

**Case:** You are a resident doctor in the emergency department of the university hospital. A 72-year old patient presents with shortness of breath and mild chest pain. You initially perform an ECG. For further assessment, you would like to carry out a focused cardiac ultrasound examination.
**Task 1: Guide** the patient through the ultrasound examination **(please ask the participants on the simulator specifically about this)**<br><br>**Task 2 + 3: Correctly obtain the standard scan planes of the FATE protocol** (including quality features, max. 90 seconds per plane), in order to get an overview of the heart function and possible underlying pathologies.<br>Freeze **each image**, **show and name the key anatomical structures**. Please do not take any measurements.

Evaluation from FATE Protocol DOPS

**Code:**	
**Simulator ☐**	**human model ☐**
**Examiner:**	

Task 1: Guide the patient through the **ultrasound examination.**

Patient confrontation/ Communication

**The following should be taken into consideration**: **(please ask the participants on the simulator specifically about this)**	**Correct**	**Upon request**	**Not at all**
▪ „Use ultrasound towels, put on gloves“	**2** □	**1** □	**0** □
▪ „Ask the patient to undress the upper body“ + „Warn the patient for the ultrasound gel“	**2** □	**1** □	**0** □
▪ Ask the patient to “lie down” + Warn the patient before applying light pressure during the examination	**2** □	**1** □	**0** □
▪ Pay attention to professional boundaries regarding intimate areas	**2** □	**1** □	**0** □

Patient confrontation Points _____/8

Task 2+3: Correctly obtain **the standard scan planes of the FATE protocol**, including quality features (max. 90 seconds per plane). Freeze **each image, show and name the key anatomical structures.**

FATE 1

Tip for the examiner: Pay attention to orientation, positioning of the transducer, coupling, image optimization, proper machine handling

Correctly obtain **the subcostal echocardiography view**, including quality features:	
▪ The 4 cardiac chambers are visualized	**2** □
▪ The leaflets of the mitral and tricuspid valve are visualized	**2** □
▪ The liver and parts of the pericardium are visualized close to the transducer	**2** □
▪ Visualization possible only with assistance	**2** □
▪ Inadequate examination despite assistance	**0** □

Correctly **demonstrate and name** the following structures:	
Right atrium	**0,5** ☐
Right ventricle	**0,5** ☐
Left atrium	**0,5** ☐
Left ventricle	**0,5** ☐

FATE 1 Points _____/ 8

Examiner´s notes:

FATE 2

Tip for the examiner: Pay attention to orientation, positioning of the transducer, coupling, image optimization, proper machine handling

Correctly obtain the **apical 4-chamber view of the heart,** including quality features:	
▪ The 4 cardiac chambers are visualized	**2** ☐
▪ The septum is demonstrated vertically	**2** ☐
▪ The mitral and tricuspid valve are demonstrated in a horizontal orientation	**2** ☐
▪ Visualization possible only with assistance	**2** ☐
▪ Inadequate examination despite assistance	**0** ☐

Correctly **demonstrate and name** the following structures:	
R + L atrium	**0,5** ☐
R + L ventricle	**0,5** ☐
Tricuspid and mitral valve	**0,5** ☐
Interatrial + interventricular septum	**0,5** ☐

Examiner´s notes:

FATE 2 Points _____/8

FATE 3

Tip for the examiner: Pay attention to orientation, positioning of the transducer, coupling, image optimization, proper machine handling

Correctly obtain the parasternal **long axis view of the heart,** including quality features:	
▪ The mitral and aortic valve lie at the same level and are demonstrated in the middle of the image	**2** ☐
▪ The interventricular septum and posterior wall of the left ventricle are demonstrated in a horizontal orientation	**2** ☐
▪ The descending aorta and the apex of the heart should be visualized	**2** ☐
▪ Visualization possible only with assistance	**2** ☐
▪ Inadequate examination despite assistance	**0** ☐

Correctly **demonstrate and name** the following structures:	
Mitral valve	**0,5** ☐
Papillary muscles	**0,5** ☐
Left ventricle	**0,5** ☐
Aortic valve	**0,5** ☐

FATE 3 Points _____/8

Examiner´s notes:

FATE 4

Tip for the examiner: Pay attention to orientation, positioning of the transducer, coupling, image optimization, proper machine handling

Correctly obtain the **parasternal short axis view of the heart at the level of the papillary muscles,** including quality features:	
▪ Round left ventricle (not egg shaped)<br>▪ Both papillary muscles, without parts of the mitral valve, are visualized<br>▪ The right ventricle is visualized (half-moon shape)	**6** ☐
▪ Only one papillary muscle can be visualized/egg shaped form of the right ventricle	**4** ☐
▪ Visualization possible only with assistance	**2** ☐

Correctly **demonstrate and name** the following structures:	
Papillary muscles	**0,5** ☐
Left ventricle	**0,5** ☐
Right ventricle	**0,5** ☐
Interventricular septum	**0,5** ☐

▪ Inadequate examination despite assistance	0 □

Examiner´s notes:

FATE 4 Points _____/8

FATE 5

Tip for the examiner: Pay attention to orientation, positioning of the transducer, coupling, image optimization, proper machine handling

Correctly obtain the **right lateral flank view at the level of the** costodiaphragmatic recess, including quality features:	
▪ The diaphragm can be visualized	2 □
▪ The costodiaphragmatic recess can be visualized	2 □
▪ Subdiaphragmatic parts of the liver are visualized	2 □
▪ Visualization possible only with assistance	2 □
▪ Inadequate examination despite assistance	0 □

Correctly **demonstrate and name** the following structures:	
Costodiaphragmatic recess	0,5 □
Lungs	0,5 □
Diaphragm	0,5 □
Liver	0,5 □

Examiner´s notes:

FATE 5 Points _____/8

FATE 6

Tip for the examiner: Pay attention to orientation, positioning of the transducer, coupling, image optimization, proper machine handling

Correctly obtain the **left lateral flank view at the level of the costodiaphragmatic recess,** including quality features:	
▪ The diaphragm can be visualized	2 □
▪ The costodiaphragmatic recess can be visualized	2 □
▪ Subdiaphragmatic parts (spleen) are visualized	2 □
▪ Visualization possible only with assistance	2 □
▪ Inadequate examination despite assistance	0 □

Correctly **demonstrate and name** the following structures:	
Costodiaphragmatic recess	0,5 □
Lungs	0,5 □
Diaphragm	0,5 □
Spleen	0,5 □

Examiner´s notes:

FATE 6 Points _____/8

**Overall performance regarding transducer manipulation according to the FATE protocol**	**Points**
**Orientation**	
▪ All scan planes can be correctly assessed/ or directly independently adjusted/ Coupling	2 □

▪ After initial difficulties, the scan plane/s can be corrected/ verbal instructions are needed	1 □
▪ Correct orientation only with assistance possible	0 □

Positioning of the transducer

▪ All scan planes can be correctly obtained/ direct transition from one plane to another	2 □
▪ After initial difficulties, the scan plane/s can be obtained/ verbal instructions are needed	1 □
▪ The transducer can be correctly positioned only with assistance	0 □

Coupling/ Transducer manipulation

▪ The transducer is covered with ultrasound gel sufficiently/ correct manipulation in all scan planes/ continuous pressure	2 □
▪ After initial difficulties, the scan plane/s can be obtained/ verbal instructions are needed	1 □
▪ No pressure and/or no ultrasound gel + uncontrolled pressure and/or insufficient transducer contact with the scanning surface	0 □

Adequate image magnification/ Image optimization/ Machine handling

▪ Independent and adequate assessment in all scan planes, good image quality (Gain, Depth, Frequency, Focus), even during the examination, left hand on the machine	2 □
▪ After initial difficulties, the scan plane/s can be obtained/ verbal instructions are needed, the examinee forgets to optimize the image during the examination, left hand not on the machine	1 □
▪ Inadequate assessment despite verbal instructions → assistance needed	0 □

**Breathing command**	
▪ Correct breathing command (as long as necessary) in all scan planes, independent performance	2 □
▪ Incomplete examination or initial difficulties, verbal instructions are needed	1 □
▪ Inadequate breathing command, even after verbal instructions	0 □
**Positioning of the patient**	
▪ In case of difficulty obtaining a certain scan plane, the patient is positioned in a left lateral position/ the patient is positioned correctly	2 □
▪ Verbal instructions are given in order to position the patient correctly	1 □
▪ Inadequate patient positioning, even after manual instructions, assistance is needed	0 □
**Duration of the examination/ Time**	
▪ Adequate, independent time management during the whole examination	2 □
▪ Sufficient after initial difficulties or after verbal instructions are given	1 □
▪ Very slow/ inadequate	0 □
**I rate the overall performance with (1 to 8 points)** (please circle)	
1 – 2 – 3 – 4 – 5 – 6 – 7 – 8	__/8

Examiner´s notes:

FATE-Protocol Overall performance points ___/22

**Total point score FATE-Protocol**<br>(Sum up: Patient confrontation + FATE 1 to 6 + Overall performance)	**___/78**
