# Supplementary material for: Simulator training in focus assessed transthoracic echocardiography (FATE) for undergraduate medical students: results from the FateSim randomized controlled trial
Source: BMC Med Educ. 2025 Jan 4;25:21. doi: 10.1186/s12909-024-06564-y (PMC11699650; doi:10.1186/s12909-024-06564-y)
Supplement: Supplementary file 7 — Supplementary Material 7 [file 12909_2024_6564_MOESM7_ESM.pdf]

DOPS Evaluation form: Detection of pathologic findings on the simulator

**Code:**	
**Examiner:**	

1) Pathology: *dilated cardiomyopathy – very severe left ventricular systolic dysfunction*

Task: Correctly obtain the ***apical 4-chamber view*** and, if necessary, the ***parasternal long axis view*** of the heart. Explain the ultrasound findings.

Demonstration of the scan planes

▪ The scan plane is demonstrated correctly/ adjusted without assistance	**2** □
▪ The scan plane is corrected upon request	**1** □
▪ Orientation only achieved with assistance	**0** □

Detection of pathologic findings

▪ The pathologic findings can be recognized/ described and reported independently	**2** □
▪ The pathologic findings can be recognized after demonstration/ can be described and reported<br>▪ **Tip for the examiner: Refer to the heart function**	**1** □
▪ The pathologic findings cannot be recognized or correctly reported	**0** □

The entire examination process is rated with (1 to 8 points)

(Orientation, Positioning of the transducer, Coupling, Image optimization, Breathing command, Positioning of the patient, Time to perform the scan and Overall performance)

1 – 2 – 3 – 4 – 5 – 6 – 7 – 8 ________/8

Total points ________/12

2) Pathology: P*ulmonary hypertension*

Task: Correctly obtain the **parasternal short axis view of the heart at the level of the papillary muscles** and, if necessary, the ***apical 4-chamber view.*** Explain the ultrasound findings.

Demonstration of the scan planes

▪ The scan plane is demonstrated correctly/ adjusted without assistance	**2** □
▪ The scan plane is corrected upon request	**1** □
▪ Orientation only with assistance possible	**0** □

Detection of pathologic findings

▪ The pathologic findings can be recognized/ described and reported independently	**2** □
▪ The pathologic findings can be recognized after demonstration/ can be described and reported<br>**Tip for the examiner: Refer to the right ventricle**	**1** □
▪ The pathologic findings cannot be recognized or correctly reported	**0** □

The entire examination process is rated with (1 to 8 points)

(Orientation, Positioning of the transducer, Coupling, Image optimization, Breathing command, Positioning of the patient, Time to perform the scan and Overall performance)

1 – 2 – 3 – 4 – 5 – 6 – 7 – 8 ________/8

Total points ________/12

3) Pathology: Cardiac *tamponade*

Task: Correctly obtain the **subxiphoid 4-chamber view of the heart,** and, if necessary, the ***parasternal short axis view at the level of the papillary muscles***. Explain the ultrasound findings.

Demonstration of the scan planes

▪ The scan plane is demonstrated correctly/ adjusted without assistance	**2** □
▪ The scan plane is corrected upon request	**1** □
▪ Correct orientation only with assistance	**0** □

Detection of pathologic findings

▪ The pathologic findings can be recognized/ described and reported independently	**2** □
▪ The pathologic findings can be recognized after demonstration/ can be described and reported **Hint for the examiner: Refer to the area surrounding the heart**	**1** □
▪ The pathologic findings cannot be recognized or correctly reported	**0** □

The entire examination process is rated with (1 to 8 points)

(Orientation, positioning of the transducer, coupling, image optimization, breathing command, positioning of the patient, time to perform the scan and overall performance)

1 – 2 – 3 – 4 – 5 – 6 – 7 – 8 ________/8

Total points ________/12

4) Pathologie: L*eft pleural effusion case 09*

Task: Correctly obtain the **left**, and if necessary, the **right lateral flank view.** Explain the ultrasound findings.

Demonstration of the scan planes

▪ The scan plane is demonstrated correctly/ adjusted without assistance	**2** □
▪ The scan plane is corrected upon request	**1** □
▪ Orientation only with assistance possible	**0** □

Detection of pathologic findings

▪ The pathologic findings can be recognized/ described and reported independently	**2** □
▪ The pathologic findings can be recognized after demonstration/ can be described and reported **Hint for the examiner: Refer to the area cranially to the diaphragm**	**1** □
▪ The pathologic findings cannot be recognized or correctly reported	**0** □

The entire examination process is rated with (1 to 8 points)

(Orientation, positioning of the transducer, coupling, image optimization, breathing command, positioning of the patient, time to perform the scan and overall performance)

1 – 2 – 3 – 4 – 5 – 6 – 7 – 8 ________/8

Total points ________/12

Examiner´s notes:

Total points for detection of pathologic findings __________/48
