# Supplementary figures and images for: Simulator training in focus assessed transthoracic echocardiography (FATE) for undergraduate medical students: results from the FateSim randomized controlled trial
Source: BMC Med Educ. 2025 Jan 4;25:21. doi: 10.1186/s12909-024-06564-y (PMC11699650; doi:10.1186/s12909-024-06564-y)

## Flow Diagram according to CONSORT

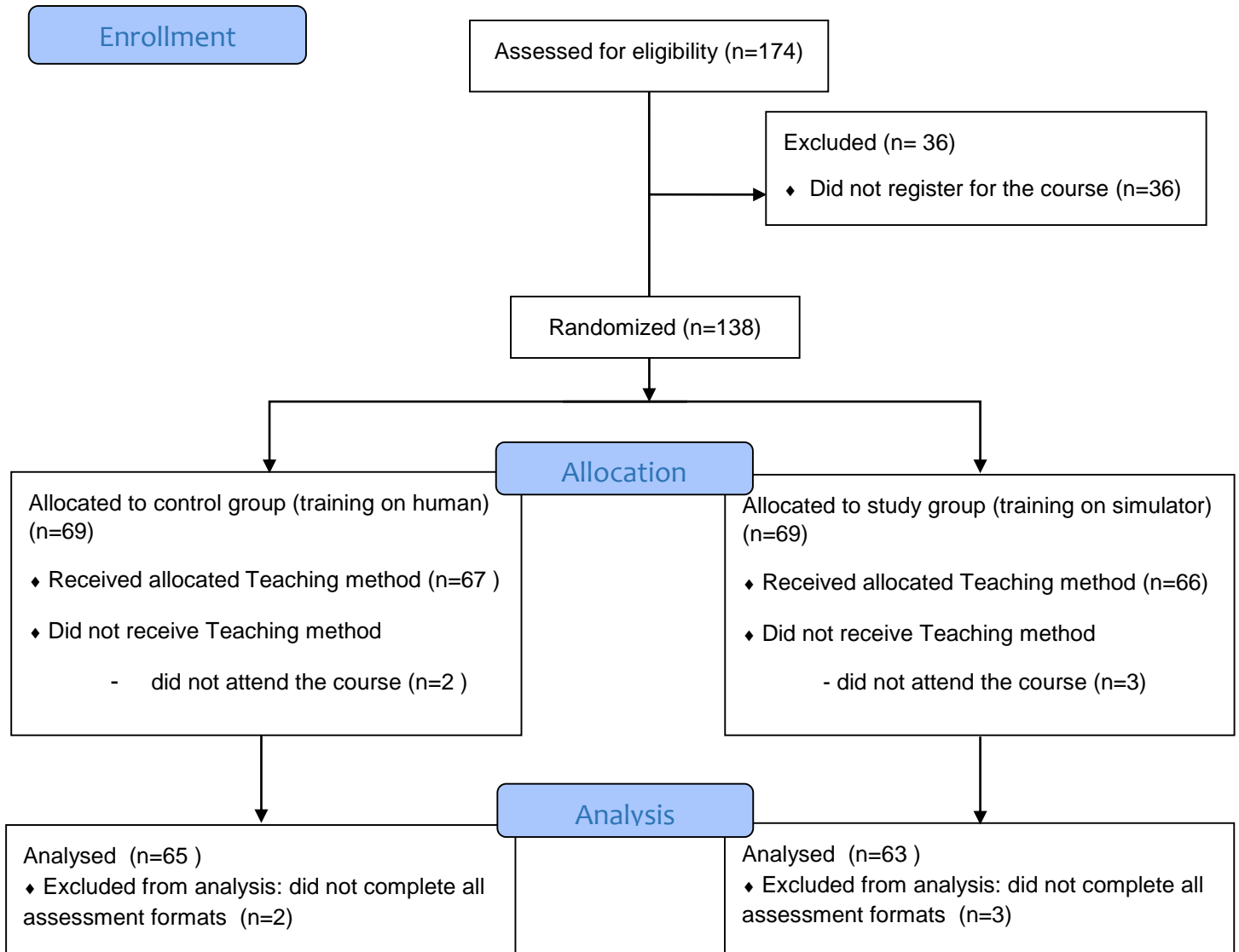

Supplement: Supplementary file 8 — Supplementary Material 8 [file 12909_2024_6564_MOESM8_ESM.pdf]
