# Supplementary material for: Simulator training in focus assessed transthoracic echocardiography (FATE) for undergraduate medical students: results from the FateSim randomized controlled trial
Source: BMC Med Educ. 2025 Jan 4;25:21. doi: 10.1186/s12909-024-06564-y (PMC11699650; doi:10.1186/s12909-024-06564-y)
Supplement: Supplementary file 9 — Supplementary Material 9 [file 12909_2024_6564_MOESM9_ESM.pdf]

Supplement 9 Motivation and Learning Objectives

	T1 study group pre (Mean±SD)	T1 control group pre (Mean±SD)	P value	T2 Study group post (Mean±SD)	T2 control group post (Mean±SD)	P value	Delta study group (p-Value by time)	Delta control group (p-Value by time)	Delta P-value
**Motivation to attend FATE**<br>*1=highly motivated - 7=not motivated*	1.6 ± 0.8	1.6 ± 1.0	0.7	1.4 ± 0.6	1.6 ± 1.0	0.26	0.2 ± 0.9<br>(0.12)	0.1 ± 1.0<br>(0.63)	0.61
**Overall: learning objectives achieved**<br>*1=completely - 7=not at all*	1.3 ± 0.5	1.2 ± 0.3	0.06	1.7 ± 0.7	1.7 ± 0.8	0.77	-0.3 ± 0.7<br>(0.003)	-0.5 ± 0.8<br>(< 0.001)	0.16
Improvement technical skills	1.4 ± 0.7	1.3 ± 0.8	0.39	1.8 ± 1.1	1.6 ± 1.0	0.31	-0.4 ± 1.0<br>(0.02)	-0.3 ± 1.2<br>(0.03)	0.8
Sono-anatomy	1.3 ± 0.6	1.2 ± 0.4	0.28	1.5 ± 0.7	1.6 ± 1.0	0.85	-0.3 ± 0.7<br>(0.02)	-0.4 ± 1.0<br>(0.002)	0.34
Practical ultrasound skills	1.1 ± 0.5	1.1 ± 0.2	0.26	1.6 ± 1.0	1.4 ± 0.7	0.29	-0.4 ± 1.0<br>(0.002)	-0.4 ± 0.7<br>(< 0.001)	0.65
Ability to perform a standardized exam	1.3 ± 0.7	1.2 ± 0.4	0.14	1.4 ± 0.7	1.5 ± 0.9	0.57	-0.1 ± 0.7<br>(0.43)	-0.3 ± 0.9<br>(0.01)	0.15
Assess pathological findings	1.5 ± 0.9	1.3 ± 0.7	0.06	1.9 ± 1.0	2.2 ± 1.3	0.06	-0.3 ± 1.1<br>(0.06)	-1.0 ± 1.4<br>(< 0.001)	0.005
Confidence in interpreting results	1.3 ± 0.7	1.1 ± 0.4	0.07	1.8 ± 0.8	1.8 ± 0.9	0.62	-0.5 ± 1.0<br>(< 0.001)	-0.7 ± 1.0<br>(< 0.001)	0.11
