# Supplementary material for: Simulator training in focus assessed transthoracic echocardiography (FATE) for undergraduate medical students: results from the FateSim randomized controlled trial
Source: BMC Med Educ. 2025 Jan 4;25:21. doi: 10.1186/s12909-024-06564-y (PMC11699650; doi:10.1186/s12909-024-06564-y)
Supplement: Supplementary file 10 — Supplementary Material 10 [file 12909_2024_6564_MOESM10_ESM.pdf]

Supplement 10 Evaluation of Course Concept and Training

	**Control Group** (Mean±SD)	**Study group** (Mean±SD)	**p-value**
**Overall personal learning experience**<br>*1=very high - 7= very low*	1.8 ± 0.7	2.3 ± 0.9	< 0.001
Acquisition of practical skills	1.8 ± 0.9	2.0 ± 1.4	0.34
Fundamental theoretical knowledge	2.2 ± 1.1	2.1 ± 0.9	0.35
Practical relevance	1.5 ± 0.7	1.7 ± 1.1	0.16
Realistic depiction of patient interaction	1.7 ± 1.0	3.5 ± 1.6	< 0.001
**Overall personal satisfaction with course and personal gain from it**<br>*1=very high - 7= very low*	2.5 ± 0.6	2.7 ± 0.7	0.05
Competence FATE protocol	2.4 ± 1.0	2.4 ± 1.1	0.97
Highly relevant for my degree/my job	1.4 ± 0.7	1.6 ± 1.0	0.09
**Overall: Desire to use Simulators**<br>*1=very high - 7= very low*	3.2 ± 1.1	3.1 ± 1.1	0.49
In addition to real patients	1.7 ± 1.2	1.6 ± 1.1	0.38
A complete replacement of real patients	5.4 ± 2.1	5.0 ± 2.3	0.37
In addition to mandatory courses in med-school (clinic)	2.4 ± 1.5	2.1 ± 1.6	0.35
In addition to mandatory courses in med-school (pre-clinic)	3.4 ± 2.0	3.7 ± 1.9	0.48
**Overall Course Evaluation**<br>*1=very high - 7= very low*	2.4 ± 0.9	2.2 ± 0.9	0.09
Course concept and structure	2.2 ± 1.1	2.0 ± 1.0	0.23
**Overall Evaluation of Tutors**<br>*1=very high - 7= very low*	1.7 ± 0.7	1.4 ± 0.7	0.03
