# Supplementary material for: Simulator training in focus assessed transthoracic echocardiography (FATE) for undergraduate medical students: results from the FateSim randomized controlled trial
Source: BMC Med Educ. 2025 Jan 4;25:21. doi: 10.1186/s12909-024-06564-y (PMC11699650; doi:10.1186/s12909-024-06564-y)
Supplement: Supplementary file 11 — Supplementary Material 11 [file 12909_2024_6564_MOESM11_ESM.pdf]

Supplement 11 Subjective Compentencies gain development

	**T1 study group pre** (Mean±SD)	**T1 control group pre** (Mean±SD)	**P value**	**T2 study group post** (Mean±SD)	**T2 control group post** (Mean±SD)	**P value**	**Delta study group (p-Value by time)**	**Delta control group (p-Value by time)**	**Delta P-value**
Overall self-assessment Echo<br>*1= very high – 7 = very low*	5.3 ±1.1	5.3 ± 1.2	0.87	2.6 ± 0.7	2.7 ± 0.7	0.28	2.7 ± 1.2<br>(< 0.001)	2.6 ± 1.2<br>(< 0.001)	0.62
Technical knowledge	5.2 ± 1.5	5.3 ± 1.8	0.77	2.5 ± 0.7	2.6 ± 1.0	0.42	2.7 ± 1.5<br>(< 0.001)	2.6 ± 1.7<br>(< 0.001)	0.9
Machine handling	5.3 ± 1.6	5.2 ± 1.7	0.81	2.7 ± 1.4	2.7 ± 1.1	0.9	2.6 ± 1.8<br>(< 0.001)	2.5 ± 1.8<br>(< 0.001)	0.76
Transducer movement	4.6 ± 1.7	4.7 ± 1.7	0.65	2.4 ± 0.9	2.4 ± 0.9	0.86	2.1 ± 1.9<br>(< 0.001)	2.3 ± 1.6<br>(< 0.001)	0.59
Spatial orientation	4.6 ± 1.5	4.7 ± 1.6	0.75	2.4 ± 1.0	2.7 ± 1.0	0.19	2.2 ± 1.7<br>(< 0.001)	2.0 ± 1.5<br>(< 0.001)	0.61
Sono-anatomy	4.8 ± 1.6	4.7 ± 1.6	0.85	2.2 ± 0.9	2.3 ± 1.0	0.59	2.6 ± 1.7<br>(< 0.001)	2.4 ± 1.7<br>(< 0.001)	0.64
Visualization of organs	5.2 ± 1.6	5.4 ± 1.6	0.48	2.4 ± 1.1	2.3 ± 0.8	0.47	2.8 ± 1.7<br>(< 0.001)	3.1 ± 1.5<br>(< 0.001)	0.27
Organ-soecific examination	5.6 ± 1.5	5.6 ± 1.5	0.82	2.6 ± 1.0	2.5 ± 0.9	0.45	3.0 ± 1.7<br>(< 0.001)	3.1 ± 1.7<br>(< 0.001)	0.52
Recognizing pathologies	6.2 ± 0.9	6.0 ± 1.2	< 0.001	2.7 ± 1.0	3.1 ± 1.2	< 0.001	3.7 ± 1.2<br>(< 0.001)	2.8 ± 1.6<br>(< 0.001)	< 0.001
Differential diagnoses	6.2 ± 1.1	6.2 ± 1.1	0.97	3.1 ± 1.3	3.7 ± 1.4	0.02	3.1 ± 1.5<br>(< 0.001)	2.5 ± 1.7<br>(< 0.001)	0.04
