# Supplementary material for: Simulator training in focus assessed transthoracic echocardiography (FATE) for undergraduate medical students: results from the FateSim randomized controlled trial
Source: BMC Med Educ. 2025 Jan 4;25:21. doi: 10.1186/s12909-024-06564-y (PMC11699650; doi:10.1186/s12909-024-06564-y)
Supplement: Supplementary file 12 — Supplementary Material 12 [file 12909_2024_6564_MOESM12_ESM.pdf]

Supplement 12 Results of Theory-Test

	**T1 study group pre** (Mean±SD)	**T1 Control group pre** (Mean±SD)	**P value**	**T2 study group post** (Mean±SD)	**T2 control group post** (Mean±SD)	**P value**	**Delta study group (p-Value by time)**	**Delta control group (p-Value by time)**	**Delta P-value**
**Overall: fundamental theoretical knowledge and normal findings** ***max. 74 points***	18.5 ± 9.5	24.4 ± 15.7	0.01	56.4 ± 6.6	57.4 ± 7.5	0.41	37.9 ± 9.2 (< 0.001)	33.0 ± 14.0 (< 0.001)	0.02
Score Fundamentals *max. 14 points*	7.8 ± 3.1	8.7 ± 4.7	0.18	9.8 ± 2.7	9.9 ± 3.3	0.88	2.1 ± 3.1 (< 0.001)	1.2 ± 3.83 (0.09)	0.17
Score Normal findings *max. 10 points*	1.1 ± 1.1	1.9 ± 2.4	0.02	8.2 ± 1.8	8.4 ± 1.6	0.55	7.1 ± 1.8 (< 0.001)	6.6 ± 2.4 (< 0.001)	0.12
Score labeling of sectional image *max. 50 points*	9.7 ± 7.9	13.8 ± 10.1	0.01	38.3 ± 4.5	39.1 ± 4.6	0.35	28.7 ± 8.2 (< 0.001)	25.3 ± 10.0 (< 0.001)	0.04
Overall: Pathologies ***max. 9 points, 1 control question***	x	x	x	4.7 ± 1.5	4.3 ± 1.6	0.16	x	x	x
