# Supplementary material for: Simulator training in focus assessed transthoracic echocardiography (FATE) for undergraduate medical students: results from the FateSim randomized controlled trial
Source: BMC Med Educ. 2025 Jan 4;25:21. doi: 10.1186/s12909-024-06564-y (PMC11699650; doi:10.1186/s12909-024-06564-y)
Supplement: Supplementary file 13 — Supplementary Material 13 [file 12909_2024_6564_MOESM13_ESM.pdf]

**Supplement 13** Results of the practical examination

| | Simulator Exam (DOPS$^{Sim}$) | | | Human Model Exam (DOPS$^{Human}$) | | | Comparison of exam performance on the simulator vs. on human models | |
|---|---|---|---|---|---|---|---|---|
| | **Control group** (Mean±SD) | **Study group** (Mean±SD) | **p-value** | **Control group** (Mean±SD) | **Study group** (Mean±SD) | **p-value** | **p-value control group** | **p-value study group** |
| FATE Overall score *max. 78 points* | 63.9 ± 8.1 | 64.1 ± 7.0 | 0.89 | 63.9 ± 8.6 | 59.4 ± 10.2 | 0.008 | 0.97 | 0.003 |
| Patient guidance/ Communication *(max. 8 points)* | 6.7 ± 1.9 | 6.8 ± 1.7 | 0.75 | 6.9 ± 1.3 | 6.6 ± 1.8 | 0.39 | 0.6 | 0.54 |
| FATE 1 *(max. 8 points)* | 6.9 ± 1.6 | 7.4 ± 1.2 | 0.04 | 6.3 ± 1.9 | 6.0 ± 2.2 | 0.39 | 0.04 | < 0.001 |
| F1 Skills *(max. 6 points)* | 5.0 ± 1.6 | 5.4 ± 1.2 | 0.06 | 4.4 ± 1.7 | 4.1 ± 2.0 | 0.41 | 0.05 | < 0.001 |
| F2 Show *(max. 2 points)* | 2.0 ± 0.3 | 2.0 ± 0.0 | 0.23 | 1.9 ± 0.4 | 1.9 ± 0.4 | 0.61 | 0.49 | 0.03 |
| FATE 2 *(max. 8 points)* | 6.6 ± 1.8 | 6.7 ± 1.5 | 0.65 | 6.2 ± 1.8 | 5.6 ± 1.9 | 0.07 | 0.17 | < 0.001 |
| F2 Skills | 4.6 ± 1.7 | 4.8 ± 1.5 | 0.47 | 4.2 ± 1.8 | 3.6 ± 1.9 | 0.09 | 0.2 | < 0.001 |
| F2 Show | 2.0 ± 0.3 | 2.0 ± 0.1 | 0.05 | 2.0 ± 0.1 | 2.0 ± 0.2 | 0.09 | 0.28 | 1 |
| FATE 3 *(max. 8 points)* | 6.1 ± 1.7 | 6.3 ± 1.7 | 0.62 | 7.0 ± 1.5 | 6.3 ± 2.0 | 0.02 | 0.003 | 0.94 |
| F3 Skills | 4.2 ± 1.6 | 4.4 ± 1.6 | 0.5 | 5.0 ± 1.4 | 4.3 ± 1.9 | 0.02 | 0.002 | 0.92 |
| F3 Show | 1.9 ± 0.2 | 1.9 ± 0.2 | 0.17 | 1.9 ± 0.2 | 1.9 ± 0.3 | 0.83 | 0.37 | 0.88 |
| FATE 4 *(max. 8 points)* | 6.3 ± 1.7 | 6.3 ± 1.3 | 0.89 | 7.0 ± 1.4 | 6.6 ± 1.5 | 0.13 | 0.01 | 0.19 |
| F4 Skills | 4.4 ± 1.5 | 4.4 ± 1.2 | 0.84 | 5.0 ± 1.4 | 4.7 ± 1.4 | 0.16 | 0.02 | 0.18 |
| F4 Show | 1.9 ± 0.4 | 1.9 ± 0.2 | 0.82 | 1.9 ± 0.2 | 1.9 ± 0.3 | 0.36 | 0.23 | 0.87 |
| FATE 5 *(max. 8 points)* | 7.6 ± 1.2 | 7.6 ± 0.9 | 0.92 | 7.5 ± 1.3 | 7.2 ± 1.5 | 0.2 | 0.78 | 0.07 |

| | | | | | | | | |
|---|---|---|---|---|---|---|---|---|
| F5 Skills | 5.7 ± 1.1 | 5.7 ± 0.9 | 0.77 | 5.6 ± 1.2 | 5.3 ± 1.4 | 0.2 | 0.76 | 0.05 |
| F5 Show | 1.9 ± 0.2 | 1.9 ± 0.2 | 0.43 | 1.9 ± 0.2 | 1.9 ± 0.2 | 0.52 | 1 | 0.85 |
| FATE 6 *(max. 8 points)* | 7.6 ± 1.1 | 7.7 ± 0.8 | 0.7 | 7.3 ± 1.3 | 7.1 ± 1.6 | 0.43 | 0.14 | 0.01 |
| F6 Skills | 5.7 ± 1.1 | 5.8 ± 0.8 | 0.62 | 5.4 ± 1.3 | 5.2 ± 1.4 | 0.63 | 0.12 | 0.01 |
| F6 Show | 1.9 ± 0.2 | 1.9 ± 0.2 | 0.65 | 1.9 ± 0.2 | 1.8 ± 0.4 | 0.15 | 0.69 | 0.38 |
| FATE Skills (F1-F6) *(max. 36 points)* | 29.5 ± 5.1 | 30.5 ± 3.6 | 0.22 | 29.6 ± 4.5 | 27.3 ± 6.2 | 0.02 | 0.91 | < 0.001 |
| FATE Show (F1-F6) *(max. 12 points)* | 11.7 ± 0.8 | 11.5 ± 0.8 | 0.36 | 11.6 ± 0.8 | 11.4 ± 0.9 | 0.1 | 0.82 | 0.36 |
| Handling *(max. 14 points)* | 10.0 ± 2.3 | 9.5 ± 2.5 | 0.21 | 9.8 ± 2.8 | 8.6 ± 2.5 | 0.009 | 0.65 | 0.05 |
| Orientation | 1.6 ± 0.5 | 1.4 ± 0.5 | 0.01 | 1.4 ± 0.6 | 1.1 ± 0.6 | 0.02 | 0.03 | 0.02 |
| Positioning | 1.4 ± 0.6 | 1.4 ± 0.6 | 0.69 | 1.3 ± 0.6 | 1.0 ± 0.6 | 0.0004 | 0.76 | < 0.001 |
| Transducer movement and manipulation | 1.5 ± 0.5 | 1.4 ± 0.5 | 0.49 | 1.5 ± 0.5 | 1.4 ± 0.5 | 0.04 | 0.60 | 0.43 |
| "Knobology" and settings (adequate depth, gain, focus and TCG) | 1.2 ± 0.5 | 1.3 ± 0.5 | 0.38 | 1.4 ± 0.6 | 1.3 ± 0.5 | 0.31 | 0.14 | 0.71 |
| Instruction of patient incl. breathing | 1.4 ± 0.6 | 1.3 ± 0.8 | 0.5 | 1.4 ± 0.6 | 1.4 ± 0.6 | 0.75 | 1 | 0.7 |
| Positioning of Patient | 1.4 ± 0.7 | 1.2 ± 0.7 | 0.2 | 1.5 ± 0.5 | 1.3 ± 0.6 | 0.05 | 0.25 | 0.42 |
| Speed of examination | 1.6 ± 0.6 | 1.5 ± 0.6 | 0.31 | 1.3 ± 0.6 | 1.3 ± 0.7 | 0.53 | 0.01 | 0.06 |
| Overall impression *(max. 8 points)* | 3.0 ± 1.0 | 3.2 ± 1.0 | 0.21 | 3.1 ± 1.4 | 3.6 ± 1.5 | 0.07 | 0.73 | 0.16 |
| | **Simulator Exam (DOPS$^{SimPatho}$)** | | | | | | | |
| **FATE Patho Overall** *max. 48 points* | 36.4 ± 5.0 | 37.4 ± 4.6 | 0.23 | x | x | x | | |
| Patho 1 *(max. 12 points)* | 8.8 ± 2.0 | 8.5 ± 1.7 | 0.49 | x | x | x | | |

| | | | | | | | | |
|---|---|---|---|---|---|---|---|---|
| P1 Skill *(max. 2 points)* | 1.7 ± 0.6 | 1.5 ± 0.6 | 0.06 | x | x | x | | |
| P1 Recog *(max. 2 points)* | 1.4 ± 0.7 | 1.5 ± 0.6 | 0.32 | x | x | x | | |
| P1 Impress. *(max. 8 points)* | 3.3 ± 1.3 | 3.4 ± 1.2 | 0.53 | x | x | x | | |
| Patho 2 *(max. 12 points)* | 8.3 ± 1.6 | 8.8 ± 1.8 | 0.08 | x | x | x | | |
| P2 Skill | 1.4 ± 0.6 | 1.5 ± 0.6 | 0.09 | x | x | x | | |
| P2 Recog | 1.4 ± 0.7 | 1.7 ± 0.5 | 0.01 | x | x | x | | |
| P2 Impress | 3.4 ± 1.2 | 3.4 ± 1.2 | 0.71 | x | x | x | | |
| Patho 3 *(max. 12 points)* | 9.6 ± 1.6 | 9.9 ± 1.6 | 0.41 | x | x | x | | |
| P3 Skill | 1.8 ± 0.4 | 1.8 ± 0.4 | 0.32 | x | x | x | | |
| P3 Recog | 1.8 ± 0.4 | 1.8 ± 0.5 | 0.64 | x | x | x | | |
| P3 Impress | 2.9 ± 1.1 | 2.7 ± 1.1 | 0.32 | x | x | x | | |
| Patho 4*(max. 12 points)* | 9.7 ± 1.3 | 10.2 ± 1.4 | 0.04 | x | x | x | | |
| P4 Skill | 1.8 ± 0.5 | 1.8 ± 0.4 | 0.24 | x | x | x | | |
| P4 Recog | 1.9 ± 0.3 | 1.9 ± 0.3 | 0.45 | x | x | x | | |
| P4 Impress | 2.9 ± 1.0 | 2.6 ± 1.0 | 0.04 | x | x | x | | |
| Skills Patho 1-4 Overall | 6.5 ± 1.3 | 6.7 ± 1.1 | 0.5 | x | x | x | | |
| Recog Patho 1-4 Overall | 6.4 ± 1.2 | 6.8 ± 1.2 | 0.08 | x | x | X | | |
